# Structural Remodeling of the Human Colonic Mesenchyme in Inflammatory Bowel Disease

**DOI:** 10.1016/j.cell.2018.08.067

**Published:** 2018-10-04

**Authors:** James Kinchen, Hannah H. Chen, Kaushal Parikh, Agne Antanaviciute, Marta Jagielowicz, David Fawkner-Corbett, Neil Ashley, Laura Cubitt, Esther Mellado-Gomez, Moustafa Attar, Eshita Sharma, Quin Wills, Rory Bowden, Felix C. Richter, David Ahern, Kamal D. Puri, Jill Henault, Francois Gervais, Hashem Koohy, Alison Simmons

**Affiliations:** 1 MRC Human Immunology Unit, MRC Weatherall Institute of Molecular Medicine, University of Oxford, John Radcliffe Hospital, Oxford OX3 9DS, UK; 2 Translational Gastroenterology Unit, John Radcliffe Hospital, Oxford, UK; 3 MRC WIMM Centre For Computational Biology, Weatherall Institute of Molecular Medicine, University of Oxford, John Radcliffe Hospital, Oxford OX3 9DS, UK; 4 Weatherall Institute of Molecular Medicine, University of Oxford, John Radcliffe Hospital, Oxford OX3 9DS, UK; 5 Wellcome Trust Centre for Human Genetics, University of Oxford, Headington, Oxford OX3 7BN, UK; 6 Kennedy Institute of Rheumatology, University of Oxford, Oxford, UK; 7 OncoResponse, Inc., Seattle, WA 98104, USA; 8 Translational Development, Celgene Corporation, Cambridge, MA, USA; 9 Novo Nordisk Research Centre Oxford, Oxford, UK

**Keywords:** inflammatory bowel disease, mesenchyme, stromal cell, crypt niche, Wnts, TNFSF14, SOX6, stratification, target discovery, single-cell RNA-seq, CyTOF

## Abstract

Intestinal mesenchymal cells play essential roles in epithelial homeostasis, matrix remodeling, immunity, and inflammation. But the extent of heterogeneity within the colonic mesenchyme in these processes remains unknown. Using unbiased single-cell profiling of over 16,500 colonic mesenchymal cells, we reveal four subsets of fibroblasts expressing divergent transcriptional regulators and functional pathways, in addition to pericytes and myofibroblasts. We identified a niche population located in proximity to epithelial crypts expressing SOX6, F3 (CD142), and WNT genes essential for colonic epithelial stem cell function. In colitis, we observed dysregulation of this niche and emergence of an activated mesenchymal population. This subset expressed TNF superfamily member 14 (TNFSF14), fibroblastic reticular cell-associated genes, IL-33, and Lysyl oxidases. Further, it induced factors that impaired epithelial proliferation and maturation and contributed to oxidative stress and disease severity *in vivo*. Our work defines how the colonic mesenchyme remodels to fuel inflammation and barrier dysfunction in IBD.

## Highlights

• Single-cell census of the colonic mesenchyme reveals unexpected heterogeneity
• Identification of the colonic crypt niche mesenchymal cell expressing SOX6 and Wnts
• Definition of fundamental aspects of mesenchymal remodeling in colitis
• Analysis of colitis-associated mesenchymal cells reveals pathogenicity drivers

## Graphical Abstract

**Figure:**
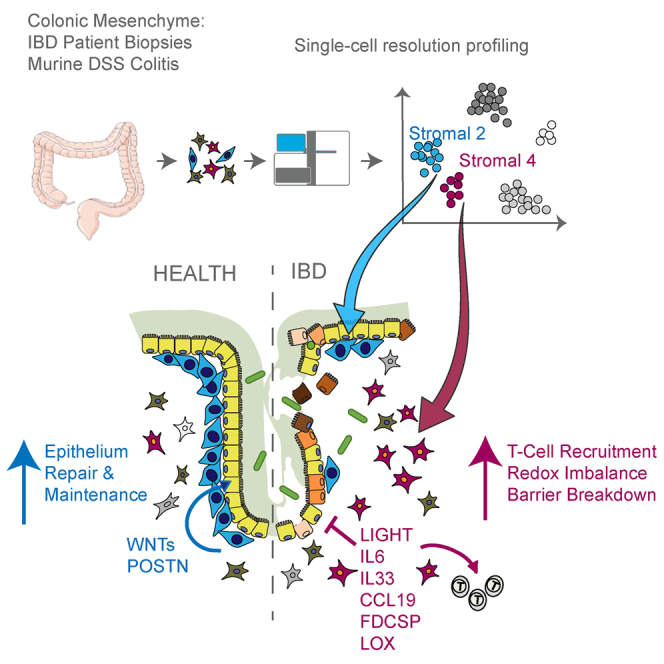

## Teaser

Single-cell profiling of human colonic mesenchymal cells identifies a colitis-associated population that expresses factors contributing to epithelial cell dysfunction and inflammation.

## Introduction

Mesenchymal cells of the intestinal lamina propria are a heterogeneous population of non-hematopoietic, non-epithelial cell types that play instrumental roles in innate immunity, immune regulation, and epithelial barrier maintenance (Nowarski et al., 2017). Their functions are impaired in inflammatory bowel disease (IBD), where they shape the inflammatory milieu, development of bowel strictures, and inflammation-associated cancers via poorly defined pathways. The major intestinal tissue stromal cell subsets are classified as fibroblasts, α smooth muscle actin (α-SMA)-expressing myofibroblasts, and perivascular pericytes (Roulis and Flavell, 2016). However, these cells express overlapping marker genes, which has prevented delineating cell-type-specific functions and ontogeny at a genetic level.

We also do not know the specific mechanisms by which colonic mesenchymal cells direct intestinal epithelial cell function. The intestinal epithelium comprises a monolayer of polarized columnar cells organized along the crypt-villus axis. Intestinal stem cells reside at the base of crypts and receive constant nourishment from the surrounding niche for maintenance, self-renewal, and differentiation. Intestinal mesenchymal cells help maintain the stem cell niche by producing Wnt agonists and antagonists, bone morphogenetic proteins (BMPs), and other molecules such as Noggin, Chordin, and R-spondins. Deregulated expression of these genes leads to colitis, impaired intestinal wound healing, or colon tumorigenesis (Koch, 2017). Although these individual molecules play defined roles in barrier maintenance, the originating cell types remain undefined. Colonic mesenchymal cells also influence intestinal mucosal immune cell function during development, inflammation, and tissue repair, shifting between immunosuppressive or pro-inflammatory states to determine the function of immune cells populating connective tissue (Bernardo and Fibbe, 2013).

Despite the growing recognition that colonic mesenchyme signals maintain epithelial barrier integrity and immune homeostasis, the identity of intestine-specific mesenchymal subtypes and the molecular attributes that regulate niche maintenance or disease remodeling have not so far been described. Single-cell RNA sequencing (scRNA-seq) has emerged as a powerful tool to define the heterogeneity of poorly classified tissue populations and disease-associated cell states. Using scRNA-seq, we identified and characterized colonic mesenchymal subsets including those that are key mediators of epithelial cell self-renewal and immune homeostasis and defined their functional contribution to inflammation in IBD patients and a murine colitis model.

## Results

### Single-Cell Profiling of Human Colonic Stromal Cells

**Figure 1:**
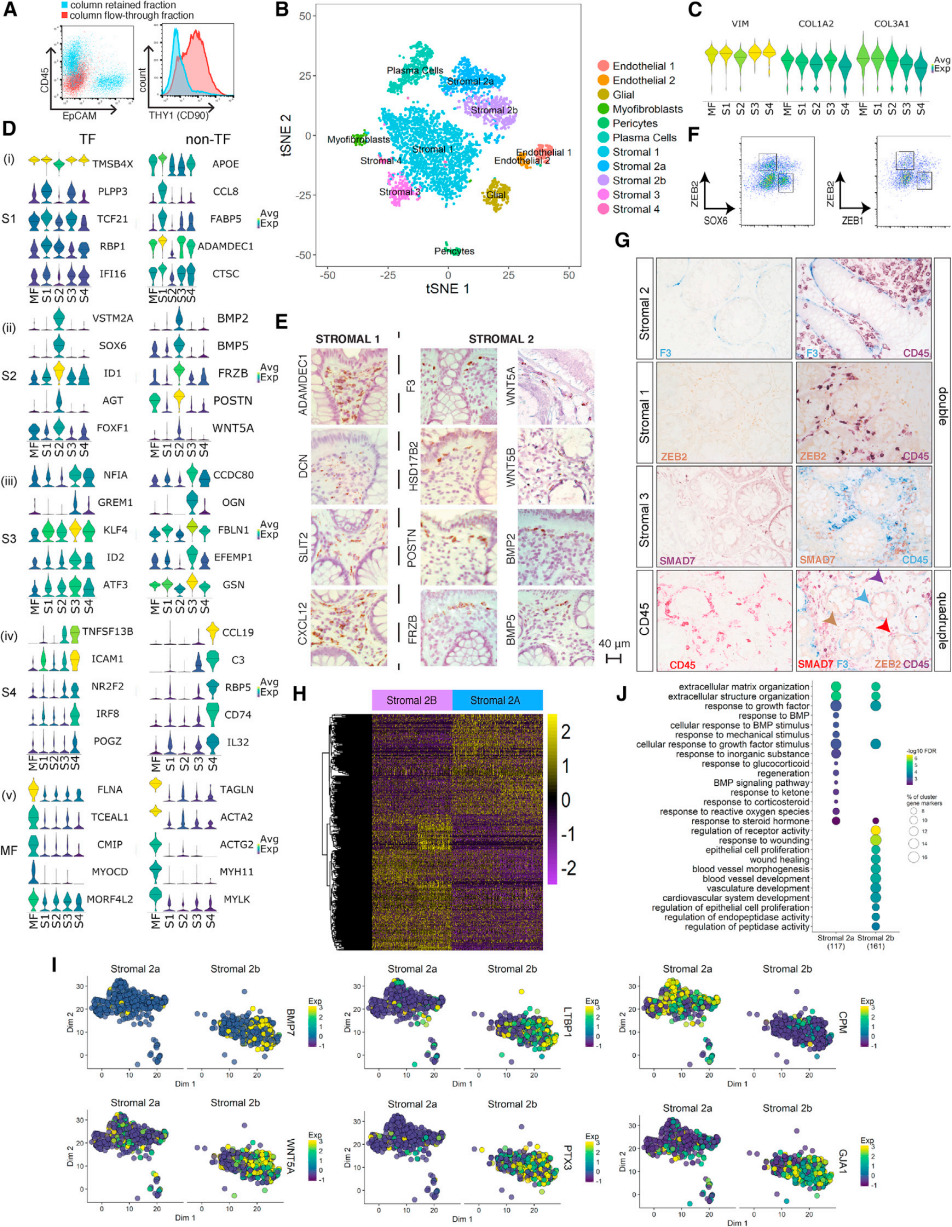
Human Colonic Mesenchymal Heterogeneity in Health (A) Flow cytometry analysis of the indicated surface markers on colonic single-cell suspensions following removal of epithelial and hematopoietic cells by MACS. Column flow-through is shown in red, and column-retained fraction is in blue. (B) t-SNE plot of the healthy human colonic mesenchyme dataset. Single cells colored by cluster annotation. (C) Violin plots for pan-fibroblast marker genes vimentin (*VIM*) and collagen types 1 and 3 (*COL1A2*, *COL3A1*) across clusters. (D) Violin plots for high-ranked transcriptional regulators and marker genes sharing GO annotation for significantly enriched terms for (i) S1 subset, (ii) S2 subset, (iii) S3 subset, (iv) S4 subset, and (v) myofibroblasts. Crossbars indicate median expression. (E) Single-molecule ISH staining of healthy human colonic tissue showing distribution of S1 markers (*ADAMDEC1*, *DCN*, *SLIT2*, and *CXCL12*) (left) and S2 markers (*F3 (CD142)*, *WNT5A*, *HSD17B2*, *WNT5B*, *POSTN*, *BMP2*, *FRZB*, *BMP5*) (right). (F) Identification of SOX6^−^ZEB2^+^/ZEB1^−^ZEB2^+^ S1 and SOX6^+^ZEB2^−^/ZEB1^+^ZEB2^−^ S2 subsets in healthy human colon. (G) Single (left) and co-staining with CD45 (right) and F3/CD142 (S2), ZEB2 (S1), and SMAD7 (S3) by IHC in colonic sections. The lower far-right panel is a quadruple stain of all 4 markers. (H) Differential expression analysis between S2a and S2b reveals 302 differentially expressed genes. (I) t-SNE plots showing examples of genes differentially expressed between S2a and S2b. (J) GO enrichment terms for S2a and S2b. See also Figures S1–S3 and Tables S1–S4.

Colonic tissue was obtained from healthy individuals undergoing screening colonoscopy or newly diagnosed IBD patients who had not received immunotherapies to avoid the effects of drug treatment on observed molecular signatures. We applied a negative selection protocol to facilitate unbiased capture of a cross-section of mesenchymal cells. We dissociated whole biopsies into single cells using magnetic-activated cell sorting (MACS) microbeads to deplete EPCAM^+^, CD45^+^, and CD235a^+^ cells. Flow cytometry analysis confirmed depleted epithelial and immune cells and enriched THY1 (CD90), a known stromal marker (Figure 1A). We then performed scRNA-seq on mesenchymal cells from 5 healthy individuals and 5 newly diagnosed IBD patients (Table S1).

### Unbiased Classification of EPCAM^–^ CD45^–^ Colonic Mesenchymal Cells in Health

**Figure S1:**
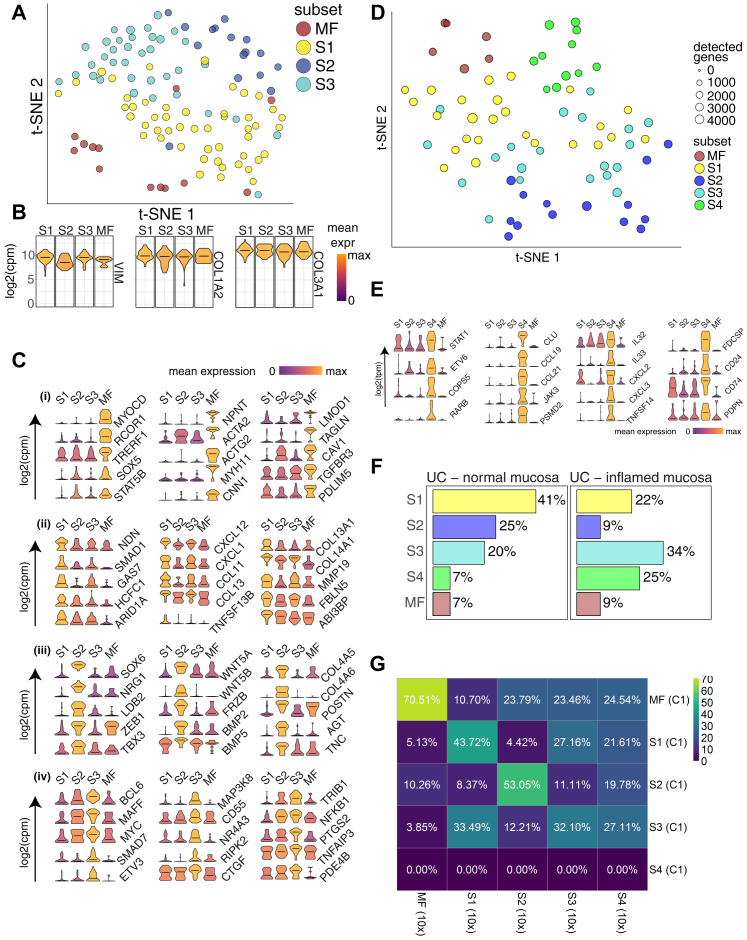
Single-Cell Profiling of Human Colonic Stromal Cells Using C1 Fluidigm Platform, Related to Figures 1 and 2 (A) t-SNE visualization of stromal cell clusters obtained from healthy human donors using the C1 Fluidigm platform. (B) Violin plots for the pan-fibroblast marker genes vimentin (*VIM*) and collagen types 1 and 3 (*COL1A2*, *COL3A1*) across clusters detected. (C) Cluster marker gene expression visualized as violin plots. (D) t-SNE visualization of stromal cell clusters obtained from IBD patients using the C1 Fluidigm platform. (E) S4 cluster marker gene visualization. (F) Cluster distribution comparison between inflamed and non-inflamed mucosa. (G) C1 healthy donor cluster marker overlap with 10x healthy donor cluster markers.

During our initial examination, we surveyed 301 cells using the C1 Fluidigm platform. Unsupervised clustering analysis revealed five distinct cell types (Figures S1A and S1D), each exhibiting similarly high expression of pan-fibroblast markers, such as the intermediate fiber vimentin and collagen types 1 and 3 (*VIM*, *COL1A2*, *COL3A1*) (Figure S1B). We designated one cluster myofibroblasts (MFs) based on high expression of contractile genes (e.g., *MYH11* and *ACTG2*), while the remaining clusters designated stromal 1–4 (hereafter S1–S4) expressed fibroblast-associated but not contractile genes and showed dissimilar transcriptional profiles and ontology enrichment (Figures S1C and S1E; Table S2).

**Figure S2:**
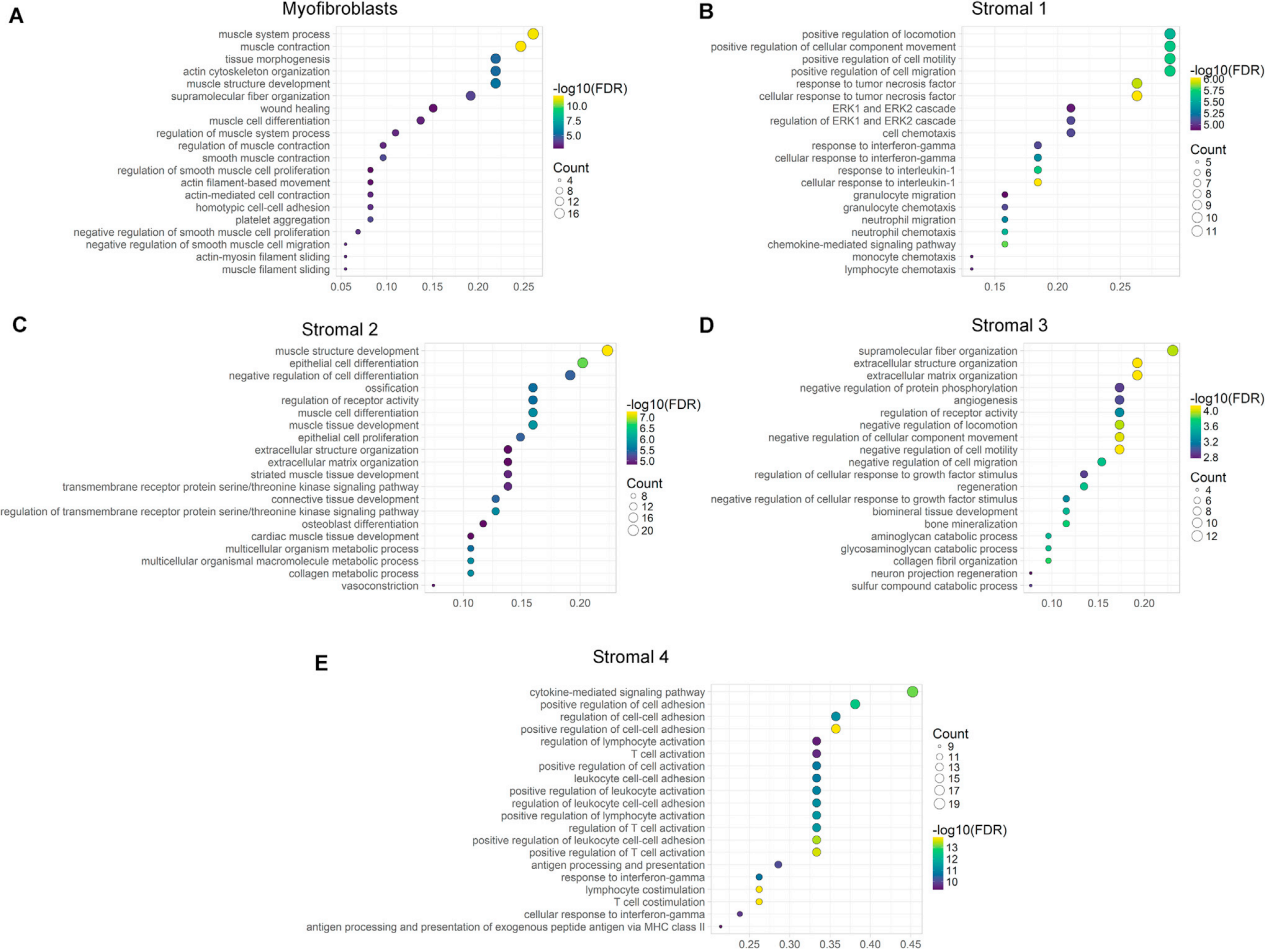
Gene Ontology Biological Process Term Enrichment Plots, Related to Figure 1 (A–E) GO enrichment plots for marker genes for (A) Myofibroblasts, (B) Stromal 1 cells, (C) Stromal 2 cells, (D) Stromal 3 Cells, and (E) Stromal 4 cells.

We then cataloged 4,378 human colonic mesenchymal cells from healthy individuals using droplet based 10x Genomics scRNA-seq to obtain a higher-resolution map. The results using this approach complemented those using the C1 Fluidigm platform. Clustering detected 11 distinct cell clusters consisting of as few as 41 cells (S4) to 1,920 cells (S1) per cluster (Figure 1B). Two clusters of endothelial cells showed marked expression of *PECAM1*, glial cells showed *S100B* expression, pericytes expressed *RGS5*, and plasma cells were identified by *SDC1* expression. We identified the remaining clusters as counterparts to fibroblast-like cell types revealed by our initial survey (Figures S1G, 1B, and 1C). Myofibroblasts were defined by gene ontology (GO) terms “muscle system process” and “muscle contraction” (Figure S2A), as well as expression of contractile genes, α-SMA (*ACTA2*) and transcription factors not previously linked to myofibroblasts, which may enable future explorations of ontogeny of these cells (Figure 1Dv).

S1 GO enrichment terms included “positive regulation of locomotion,” “response to tumor necrosis factor,” and “ERK1 and ERK2 cascade” (Figure S2B). Examples of preferentially induced genes included *APOE*, *CCL8*, *FABP5*, *ADAMDEC1* (Figure 1Di). Stromal sub-populations showed enrichment for genes annotated with “extracellular matrix”-related GO terms (Figure S2), a central fibroblast function, but they differed in the expression of specific forms of collagen. S1 enriched for non-fibrillar collagens (*COL14A1*, *COL15A*) and elastic fibers (*FBLN1*, *FBLN2*, *FBLN5*, *EFEMP1*, *FN1*), while S2 showed specific expression of sheet collagens (*COL4A5*, *COL4A6*) that are key constituents of the epithelial basement membrane, which suggests S2 may play a role in epithelial barrier maintenance (Figure S1C; Table S3).

S2, marked by expression of the transcription factor *SOX6* (Figure 1Dii), consisted of two similar sub-clusters designated 2a and 2b (Figure 1B). S2 had high expression of transforming growth factor β (TGF-β) superfamily ligands (*BMP2* and *BMP5*), non-canonical Wnt ligands (*WNT5A* and *WNT5B*), and the secreted Wnt antagonist *FRZB* (Figures 1Dii and S1C). WNT5A is essential for epithelial reconstitution after injury via a mechanism that involves potentiation of TGF signaling (Miyoshi et al., 2012). S2 also expressed high levels of periostin (*POSTN*) (Figure 1D, ii), which is essential for tissue repair but can also promote tumorigenesis (Bao et al., 2004). The combination of factors secreted by S2 indicates it may contribute to epithelial stem cell proliferation and differentiation and constitute an important mesenchymal niche cell.

S3 GO enrichment included “supramolecular fiber organization” and “extracellular cluster organization’ (Figures S2D and 1Diii), whereas S4, which consisted of very few cells in healthy mesenchyme, showed enriched GO terms, including “cytokine signaling pathway,” “positive regulation of cell adhesion,” and “T cell activation” (Figure S2E).

We next sought to define the tissue distribution of these newly identified mesenchymal subsets using immunohistochemistry (IHC) and single-molecule *in situ* hybridization (sm-ISH). We detected S1 markers (*ADAMDEC1*, *DCN*, *SLIT2*, *CXCL12*) in mesenchymal cells distributed throughout the lamina propria, while S2 markers (*F3* [*CD142*], *WNT5A*, *WNT5B*, *BMP2*, *BMP5*, *FRZB*, *POSTN*, *HSD17B2*) were restricted to a smaller sub-population in close proximity to the epithelial monolayer. This precise anatomical localization, in combination with their epithelial regeneration-associated gene expression profile, indicates a likely role for S2 in directing the function of epithelial progenitors and epithelial homeostasis (Figure 1E). We further validated the existence of these new populations by flow cytometry analysis of fibroblasts from healthy human colonic tissue. Here, we distinguished distinct populations of SOX6^−^ZEB2^+^/ZEB1^−^ZEB2^+^ S1 and SOX6^+^ZEB2^−^/ZEB1^+^ZEB2^−^ S2 cells (Figure 1F). We quantified the spatial segregation of stromal subset markers by IHC and co-staining colonic tissue sections with antibodies detecting a key marker protein from each new subset together with CD45 to distinguish immune cells. Figure 1G shows distinct segregation of the three proteins marking the novel subsets from each other and immune cells in a quadruple stain in healthy human colonic tissue.

We examined differentially expressed genes between S2a and S2b, the crypt niche population. 302 marker genes differentiated these closely related sub-clusters (Figure 1H; Table S4). Examples of genes segregating S2 into sub-clusters a and b included *BMP7*, *WNT5a*, *CPM*, *PTX3*, *LTBP1*, and *GJA1* (Figure 1I). We further examined the S2a and S2b sub-clusters by comparing their over-represented GO terms in positive marker genes for S2a and S2b sub-clusters (Figure 1J). This analysis revealed S2a expressed genes with GO relating to “BMP signaling and response,” whereas S2b expressed factors relating to “response to wound healing” and “regulation of epithelial cell proliferation.”

Overall, our data identified new and distinct colonic mesenchymal subsets with specific functional properties that exhibited unique marker gene expression and anatomical location within the lamina propria. In particular, we identified a putative intestinal crypt niche mesenchymal cell (S2a and S2b) hallmarked by gene expression required for epithelial progenitor cell function and proliferation.

### Creating a Mesenchymal Atlas of Stromal Cells from Ulcerative Colitis Patients

**Figure 2:**
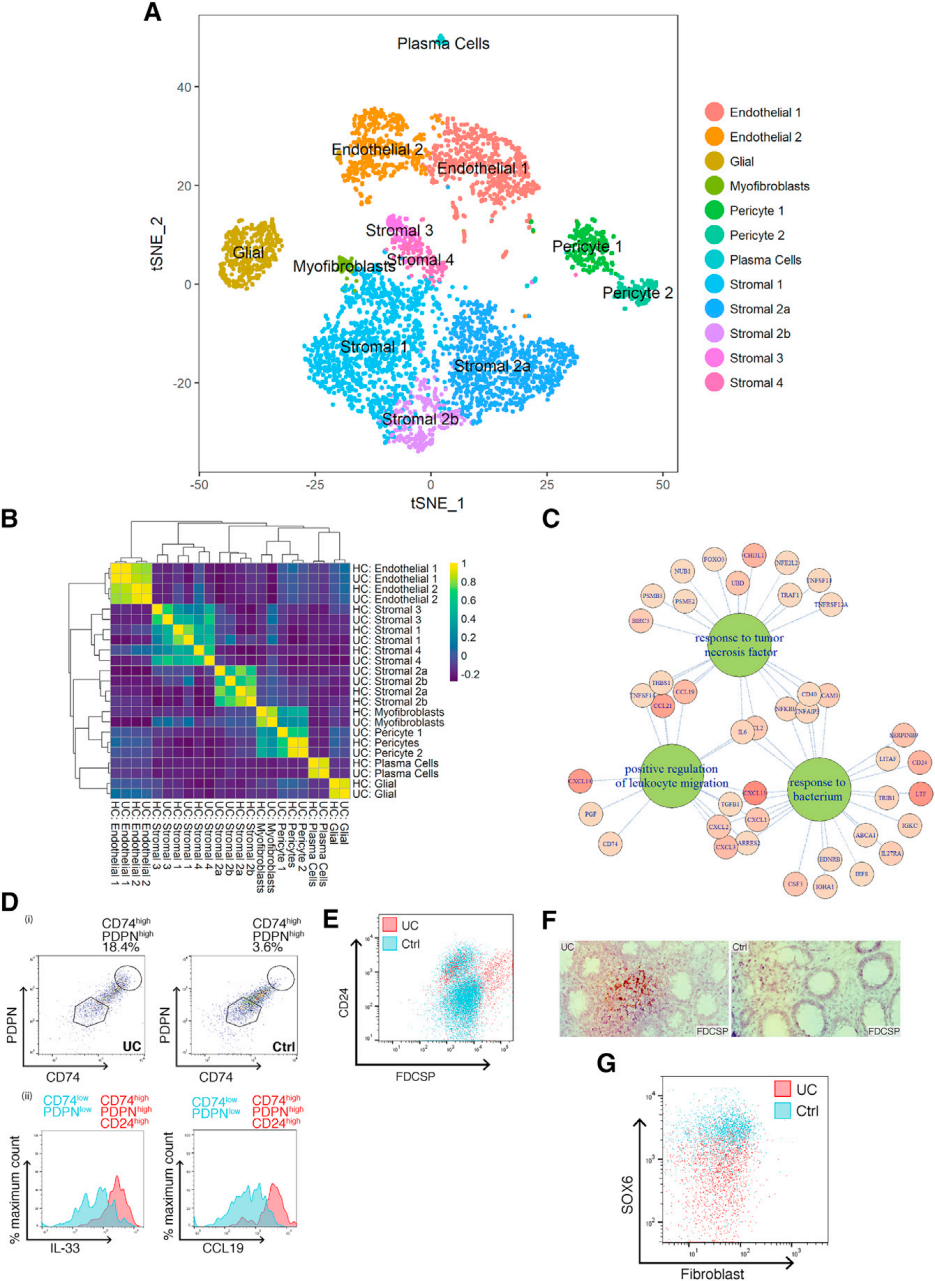
Colonic Mesenchymal Plasticity in IBD (A) t-SNE plot of UC colonic mesenchyme dataset. Single cells colored by cluster annotation. Descriptive cluster labels are shown. (B) Human healthy and UC cluster marker gene overlap correlation heatmap. (C) Selected enriched (FDR < 0.01) GO terms of UC S4 mesenchymal population marker genes. (D) (i) Flow cytometry analysis of CD74 and PDPN expression on colonic stromal cells from Ctrl (right) or UC (left) donors. (ii) Comparison of intracellular CCL19 and IL-33 levels in CD74^high^PDPN^high^CD24^high^ cells (red) versus the corresponding CD74^low^PDPN^low^ subset (blue) in inflamed UC colonic tissue. (E) Flow cytometry analysis of FDCSP^high^ and CD24^high^ colonic stromal cells from Ctrl (blue) or UC (red). (F) Single-molecule ISH staining of *FDCSP* in Ctrl or UC colonic tissue sections. (G) Flow cytometric analysis of SOX6 expression in Ctrl (blue) or UC (red) colonic stromal cells. See also Figures S1 and S3 and Tables S1 and S5.

To uncover the role of our newly identified mesenchymal subsets in IBD, we investigated changes in their composition and gene expression at the single-cell level in patients with ulcerative colitis (UC). scRNA-seq of UC colonic mesenchyme revealed 12 distinct clusters of cells. A random forest classifier trained using the data from healthy patients guided the identification of corresponding UC cell clusters. We readily identified the same clusters as detected in healthy mucosa, except an additional small cluster of pericytes (Figure 2A). A healthy and UC cluster marker gene overlap correlation heatmap showed major cell types were preserved in UC (Figure 2B). We identified changes in the proportions of various clusters including expansion of endothelial cells and pericytes. Within the stromal subsets, we observed expansion of S4 that was barely detectable in the healthy mesenchyme (Figure 2A). This finding is consistent with our preliminary data using the C1 Platform (Figures S1A and S1D; Table S5).

We further explored the nature of S4. GO enrichment terms for this subset in UC included “response to tumor necrosis factor,” “positive regulation of leukocyte migration,” and “response to bacterium” (Figure 2C). Highly ranked S4 markers included fibroblastic reticular cell (FRC)-associated genes, lymphocyte trafficking cytokines (*CCL19* and *CCL21*), T cell co-stimulatory TNF-superfamily ligand (*TNFSF14/LIGHT)*, the major histocompatibility complex (MHC) class II invariant chain (*CD74*), the molecular chaperone clusterin (*CLU*), CD24, and interleukin-33 (IL-33) (Figures 2C and S1; Table S5). So, scRNA-seq identified expansion of a novel stromal population enriched for pro-inflammatory and FRC genes in UC.

**Figure S3:**
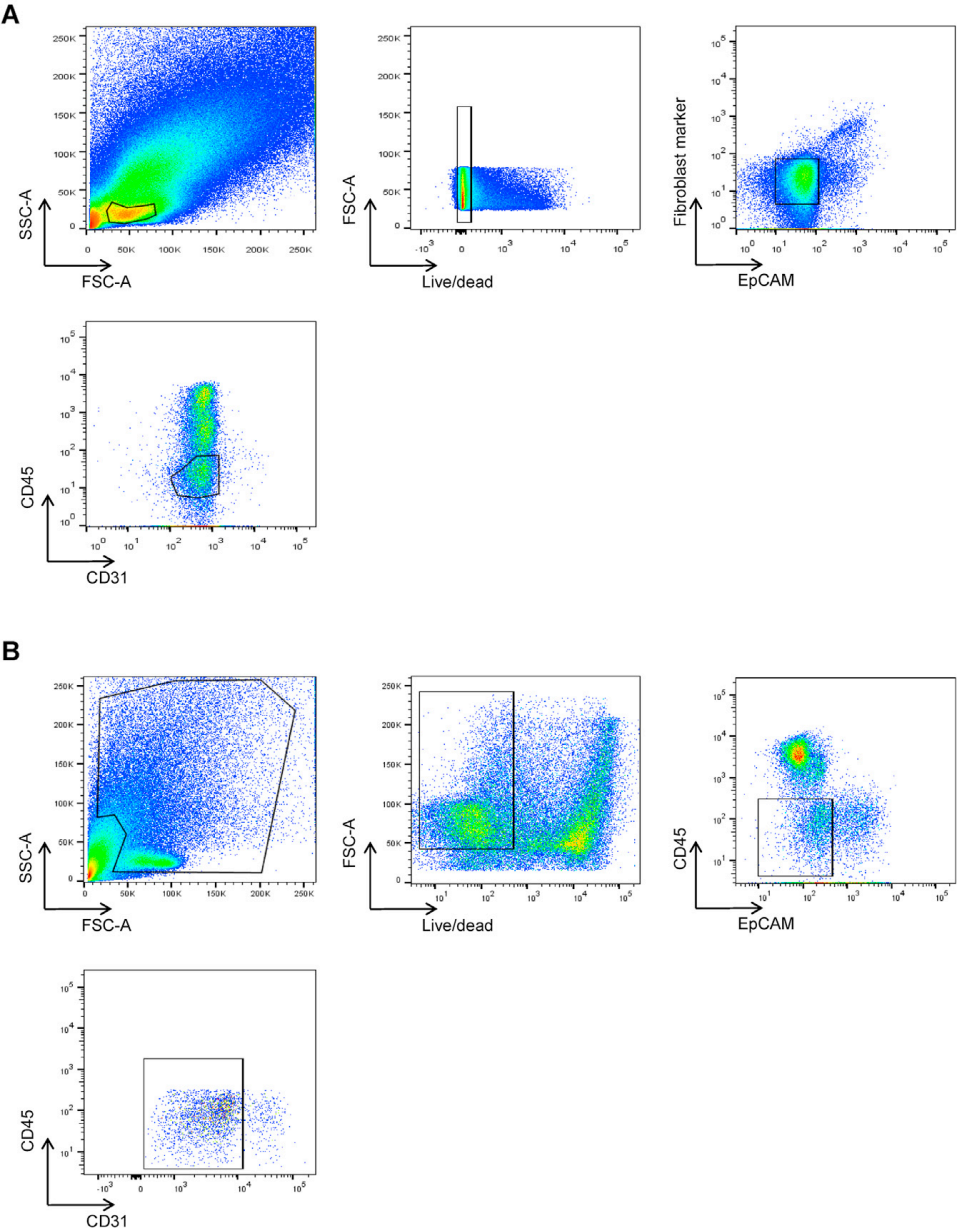
Flow Cytometry Gating Strategies on Intestinal Stromal Cells from Human Colonic Biopsies, Related to Figures 1 and 2 Representative gating strategies for analyses of EpCAM^-^CD45^-^CD31^-^ human colonic stromal subsets. (A) Gating strategies for the detection of nuclear targets. (B) Gating strategies for the detection of cytoplasmic targets.

Next, we investigated whether we could detect S4 cells at the protein level in colonic tissue samples from IBD patients. We stained colonic cell suspensions derived from UC patients and healthy controls with antibodies to predicted S4 markers. Colonic stromal cells from active UC showed enriched S4 proteins CD74 and PDPN (Figure 2D, i). Cells expressing S4 markers CD74, CD24, and PDPN showed increased CCL19 and IL-33 expression (Figure 2D, ii). Flow cytometry analysis confirmed the expansion of a FDCSP^high^, CD24^high^ population of stromal cells in inflamed UC tissue (Figures 2E and S3). We also found increased FDCSP expression within the lamina propria of inflamed UC tissue sections by sm-ISH (Figure 2F).

In addition to expansion of S4 in UC, we observed a decrease in SOX6^+^ S2 cells in biopsies from inflamed UC colonic tissue when compared to healthy controls (Figure 2G), also observed in our preliminary C1 data (Figure S1F). Taken together, these findings chart the nature of mesenchymal plasticity in human IBD, demonstrating dysregulation of the crypt niche S2 population, which presents a novel feature of barrier dysfunction in UC. Simultaneously, we observed the emergence of activated S4 equipped to mobilize the immune response and drive tertiary lymphoid follicle formation.

### Comparing Murine and Human Colonic Mesenchymal Stromal Cells

**Figure S4:**
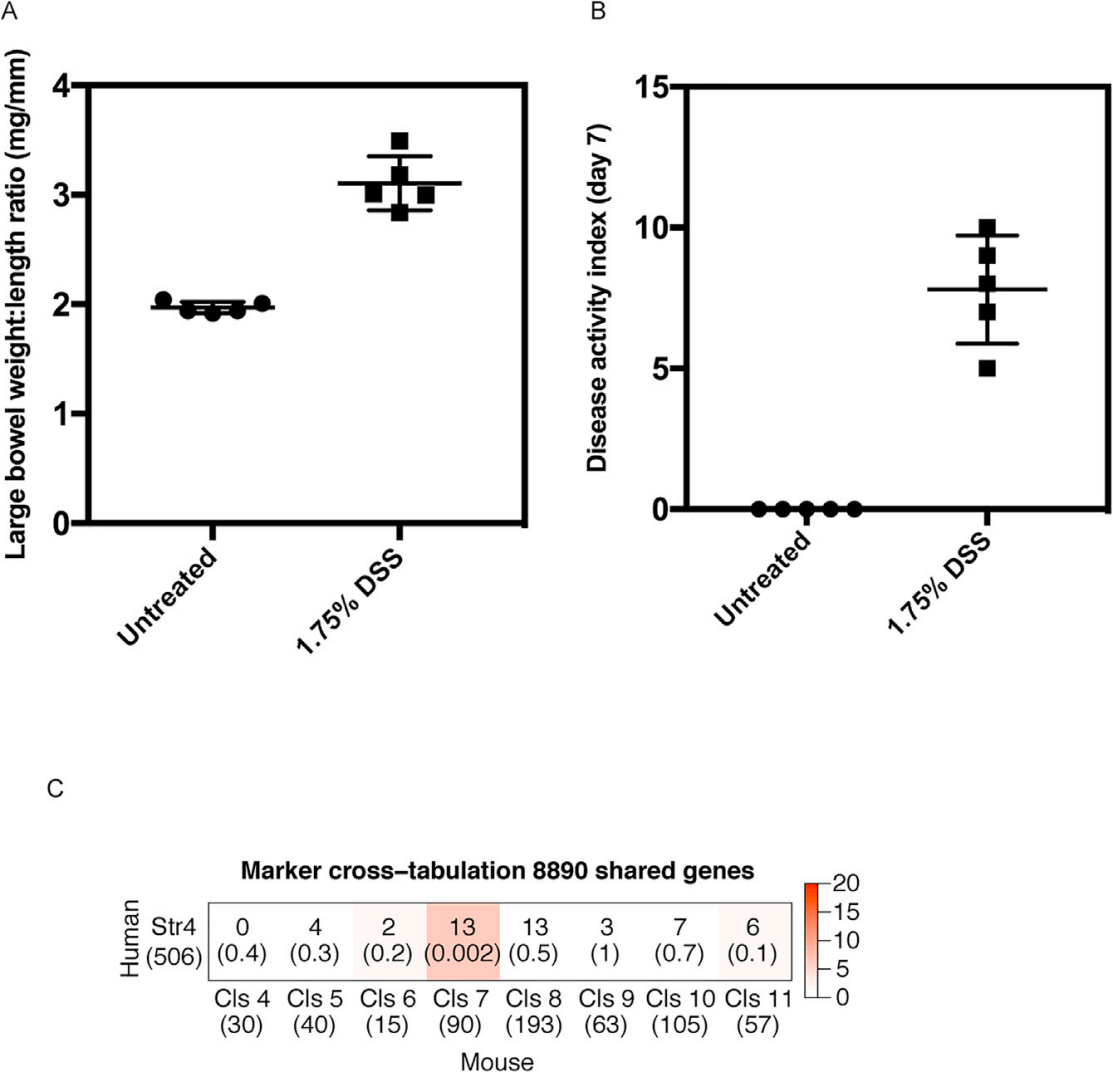
Murine DSS Challenge, Related to Figures 3 and 4 Colonic stromal cells were isolated from age and sex matched Ctrl mice or mice treated with DSS for 7 days. (A) Ratio of large bowel weight to length by treatment group. Measurements were made post-mortem on study day 7. (B) A composite score of in-life disease activity measures (comprising weight loss, diarrhea and rectal bleeding) for all treatment groups. Group means are indicated (cross-bars). (C) An immunologically specialized fibroblast subset analogous to human Stromal 4 is identified in the murine DSS model. Cross-tabulation of human Stromal 4 marker genes against marker genes for the 8 clusters of fibroblast-like cells identified in the DSS dataset. The number of shared markers and p value (Fisher’s Exact Test) are shown. Color scale −log(p value).

Dextran sodium sulfate (DSS) colitis is a commonly used colitis model that leads to a pro-inflammatory phenotype with parallels to human IBD. We predicted similar mesenchymal heterogeneity might exist in murine intestine in health and following DSS challenge. We examined this using the 10x Genomics platform. We divided male C57BL/6 mice into control and treatment groups and administered a DSS challenge (Figures S4A and S4B). Stromal cells were enriched from the entire colon by MACS depletion of epithelial and immune cells. Following control cell removal and quality control (QC), 7,171 single cells remained in the analysis (3,817 healthy, 3,354 DSS).

**Figure 3:**
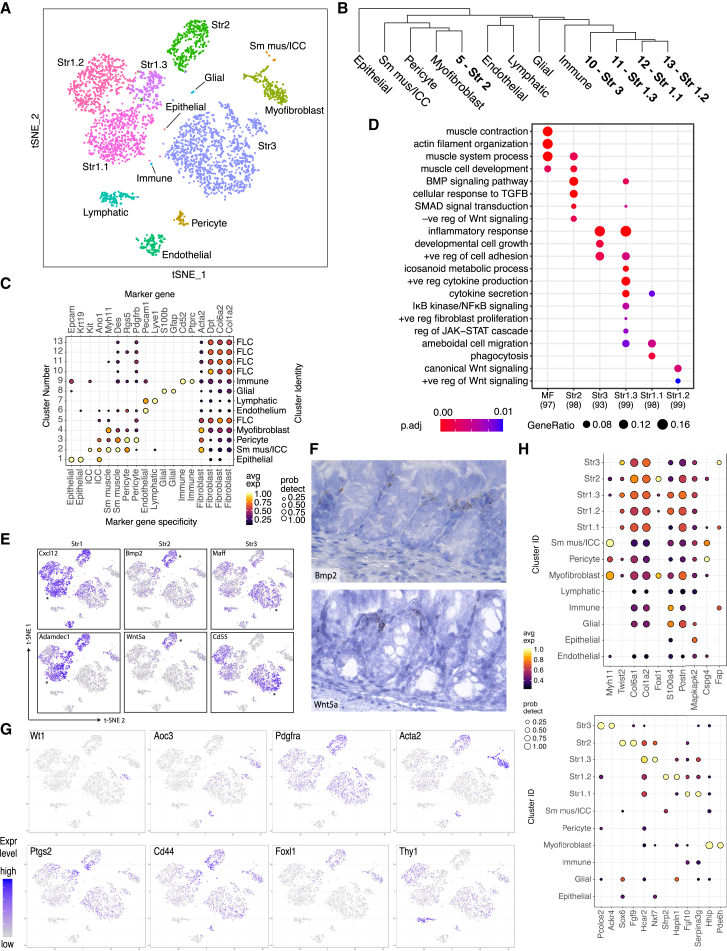
Phylogenetic Tree and Identity of Murine Colonic Mesenchymal Cells in Health (A) t-SNE plot of murine healthy colonic mesenchyme dataset. Single cells colored by cluster annotation. (B) Phylogenetic tree of murine clusters representing inter-cell distances between the average cells for each cluster in gene expression space. (C) Dot plot showing expression of canonical marker genes against detected clusters. Circle size represents the within-cluster probability of gene detection. Fill color represents the normalized mean expression level. Cell-type specificity for each marker is indicated (color bar). Numeric cluster identifiers and corresponding inferred cell types shown (left and right y axis labels). (D) Selected GO terms showing significant enrichment among top marker genes for stromal clusters. The number of markers identified for each cluster indicated (x axis). Circle size corresponds to the proportion of markers annotated to a given term, while the fill color indicates the adjusted p value. (E) t-SNE expression plots of human fibroblast subset markers in the murine dataset. Cells colored by normalized expression of indicated marker genes. The murine cluster with the highest mean expression is indicated (^∗^). Left, S1; middle, S2; and right, S3 markers. (F) sm-ISH localization of S2 genes (*Bmp2* and *Wnt5a).* (G) Expression of historical murine colonic fibroblast markers segregated across novel mesenchymal clusters identified by scRNA-seq. (H) Candidate molecular markers for future subset characterization. Specificity of candidate marker genes (x axis) for detected fibroblast subsets. Top: Existing markers. Bottom: New markers showing high subset specificity in this dataset. Circle size represents the within-cluster probability of gene detection. Fill color represents normalized mean expression level. See also Figure S4 and Tables S1 and S6.

Clustering cells from healthy mice revealed 13 distinct clusters (Figure 3A and 3C). We readily identified clusters showing specific expression of epithelial (*Epcam* and *Krt19*), pericyte (*Rgs5* and *Pdgfrb*), vascular endothelial (*Pecam1* / *Cd31*), lymphatic endothelial (*Lyve1*), and glial (*S100b* and *Gfap*) and hematopoietic cell markers (*Cd52* and *Ptprc* / *Cd45*) (Figure 3C). Cluster 2, a small cluster of 32 cells, expressed markers associated with enteric smooth muscle (*Myh11* and *Des*) and interstitial cells of Cajal (ICCs) (*Kit* and *Ano1*). Further examination of this cluster revealed its composition was two distinct sub-clusters consistent with ICCs and smooth muscle cells, respectively (Figure 3A). Other low-abundance clusters included enteric glial cells (14 cells) and pericytes (67 cells) (Figure 3A). The remaining 6 cell clusters (4–5, 10–13), comprising 3,391 cells or 89% of the dataset were fibroblast-like cells (FLCs) characterized by expression of the pan-fibroblast markers such as *Dpt*, *Col6a2*, and *Col1a2* (Figure 3C). Clusters 4 and 5 also showed *α-Sma* expression, while only cluster 4 showed significant expression of smooth muscle myosin (*Myh11*) (Figure 3C). We readily identified these six populations as putative counterparts to the stromal cell populations in our human data by cluster marker expression (Table S6).

Figure 3B shows a phylogenetic tree of healthy murine colonic mesenchymal clusters and Figure 3D differential GO enrichment between these clusters, showing divergent functional specialization. In addition, we observed GO enrichment for myofibroblasts, which enriched predominantly for contractile processes, while the S2 group enriched for TGF-β responses and BMP signaling. The enrichment for “ameboidal-type cell migration” seen in S1.1 and S1.3 may represent migratory properties of colonic stroma (Brown et al., 2007). Murine clusters were assigned identifiers based on the human cluster to which they were most similar. In the case of S1, where a one-to-many relationship was observed, decimal point identifiers were appended to denote sub-clusters. Murine cluster 11 overlapped with human S1 and S3 but showed a close phylogenetic relationship to the other S1 clusters, so it was termed S1.3 (Figure 3B).

Overall, key subset-specific marker pairs identified from the human data demonstrated correlated expression in the mouse (Figure 3E). Murine S2 markers localized to the same peri-epithelial anatomical location as their human counterparts (Figure 3F). We examined how expression of previously reported murine colonic fibroblast markers segregated across mesenchymal subsets identified by scRNA-seq (Figure 3G). The mesothelial marker *Wt1* showed expression within a small, localized subpopulation of S3 cells (Wilm et al., 2005). The myofibroblast marker *Aoc3* was detected predominantly in the myofibroblast and pericyte clusters (Hsia et al., 2016). *Ptgs2* and *Cd44* were detected it at the interface between S1.3 and S2 groups. *Pdgfrα* expression was maximal in S2 though present in all subsets. α-SMA (*Acta2*) was maximally expressed in smooth muscle with progressive reductions in expression in the myofibroblast, pericyte, and S2 groups. This is consistent with our experimental observation of distinct populations of PDGFRα^+^ and α-SMA^+^ cells, likely S2 and myofibroblasts, in the pericryptal sheath (Kurahashi et al., 2013). *Foxl1* expression, which identifies mesenchymal cells contributing to the epithelial stem cell niche, was localized to myofibroblasts and S2 (Aoki et al., 2016). Lymphatic endothelial cells showed maximal expression of *Thy1* (*Cd90*), with the S1.2 group showing intermediate expression of this marker (Pinchuk et al., 2008).

We also examined expression of genes utilized for existing stromal Cre recombinase models—*Myh11* targeted smooth muscle and myofibroblasts, *Cspg4* (*Ng2*) pericytes, and *Fap* S3. By ranking the scRNA-seq-derived subset marker genes by specificity, we could propose novel candidates for construction of selective models targeting individual stromal subsets (Figure 3H).

### Inference of Stromal Subtype Relationship by Diffusion Pseudo-time

**Figure 4:**
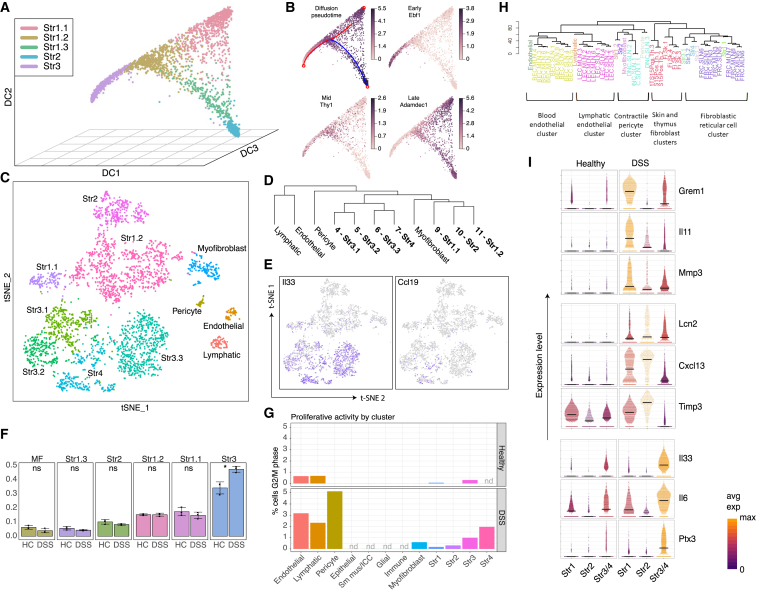
Murine Colonic Stromal Cells in Colitis (A) Diffusion component plot for colonic stromal cells from healthy mice. Individual points represent single cells colored by cluster annotation. (B) Projection of pseudo-time (top left) and selected gene expression onto diffusion map. (C) t-SNE projection of 3,354 single cells derived from 3 mice following DSS challenge. A random forest classifier trained using the healthy dataset classified cells from DSS-challenged mice. Identities of clusters in the DSS dataset were inferred and are colored by cluster annotation. (D) Phylogenetic tree and identities of murine stromal cell clusters in DSS colitis. Phylogenetic tree represents inter-cell distances between the average cells for each cluster in gene expression space. (E) t-SNE representation of the DSS dataset showing expression of S4 marker genes *Il33* and *Ccl19*. (F) Increased relative abundance of the S3 subset in DSS colitis. The size of each fibroblast cluster (column facets) expressed as a proportion of the total number of cells was compared across three biological replicates for healthy controls (HC) and DSS-challenged mice (DSS). Individual data points, mean, and SD shown. DSS challenge significantly increased the fraction of S3 cells (p = 0.02). (G) Fibroblast subsets show differential proliferative activity on DSS challenge. Cell-cycle-phase annotation for the healthy and DSS datasets using a pre-trained murine cell-cycle classifier (cyclone, “pairs” method). Percentages of cells in G2M phase by cluster (nd, no equivalent cluster detected in dataset). (H) Phylogenetic tree showing similarity between murine colonic mesenchymal stromal subsets and murine stroma obtained from lymphoid tissue. (I) Stromal subsets show differential responses to DSS challenge. Violin plots for indicated genes significantly induced on DSS challenge in S1–3. Individual cells represented as points. Color scale reflects row-normalized mean expression. Crossbars indicate cluster median expression. See also Figure S4 and Tables S1, S7, and S8.

The complete transcriptome data from healthy stromal cells allowed us to interrogate the relationships between these cells. We conducted diffusion pseudo-time analysis to order mesenchymal cells in pseudo-time to infer their developmental trajectories. Non-fibroblast cell types were first removed from the control dataset as these were considered unlikely to form part of the same developmental hierarchy. We also removed the myofibroblast cluster, as this clustered separately from the remaining fibroblasts in the diffusion map space, and intermediate forms were not observed at this sampling density. The remaining clusters (S1.1, S1.2, S1.3, S2, and S3) produced the branched structure shown in Figure 4A. While any of the three vertices (populated by S1.1, S2, and S3 cells) could represent the origin, S3 was considered the most likely candidate given its expansion and proliferative activity on DSS challenge. So, we calculated diffusion pseudo-time from this point. This placed S2 and S1.1 as fully differentiated states. S1.2 appeared an intermediate state between the crypt niche and parenchymal fibroblast with S1.3 lying between this intermediate state and the crypt niche. Using this model, we could identify genes, such as *Ebf1*, *Thy1*, and *Adamdec1*, predicted to show sequential induction (Figure 4B).

### Mesenchymal Plasticity in DSS Colitis

We next examined the nature of mesenchymal remodeling following DSS challenge. 3,354 mesenchymal cells were sequenced from DSS-challenged mice, and a random forest classifier was then trained using the healthy dataset. We cross-tabulated the results of clustering and random forest classification to determine the identities of the DSS clusters (Figure 4C). Endothelial, lymphatic, pericyte, myofibroblast, S1.1, and S2 clusters were clearly identified (Tables S7 and S8). Cells comprising DSS cluster 11 were mostly classified as S1.2, albeit with a minority classified as S1.1. DSS clusters 4–7 were all classified as S3 (Figure 4D), suggesting that heterogeneity within the S3 group increased in the presence of DSS-driven inflammation. We examined whether DSS challenge led to the emergence of a population equivalent to the activated S4 population observed in human IBD using cross-tabulation. There was significant overlap between the murine orthologs of S4 markers and murine colonic stroma DSS cluster 7 (Figure S4C). Figure 4D shows a phylogenetic tree and identities of murine stromal cell clusters in DSS colitis. We assigned S3 sub-clusters decimal suffixes with the exception of cluster 7, which we labeled as S4 on the basis of its overlap with the corresponding human subset (Figure S4C). Among the shared marker genes identified were the FRC-associated chemokine *Ccl19* and the IL-1 family alarmin *Il33* (Figure 4E).

Using the random forest classifier, we quantified changes in stromal subtype composition associated with DSS challenge. We found a significant increase in the relative abundance of S3 cells from 34% to 47% of the dataset (Figure 4F). This could arise from differential rates of proliferation or cell loss among other stromal subtypes. To investigate the former, we utilized a cell-cycle classifier to annotate the predicted cell-cycle stage of each cell in the dataset. We determined cell-cycle scores for G1 and G2/M phases for each cell using a panel of gene pairs known to exhibit cell-cycle-stage-specific expression in murine cell lines (Scialdone et al., 2015). The majority of colonic fibroblast-like cells in both treatment groups were in the G0/G1 phase, as expected. However, there was an increase in G2/M annotated cells on DSS challenge. Pericytes and vascular and lymphatic endothelial cells showed the highest proliferative activity. Among fibroblast-like clusters, the highest G2M proportions were observed in the S3 and S4 subsets, indicating proliferation accounts at least partially for their increased abundance following DSS challenge (Figure 4G).

We next determined the identity of the S4 population that expands in both DSS colitis and human IBD. Using published gene expression data to compare the murine colonic mesenchymal subsets we identified with murine blood or lymphatic endothelial cells, pericytes, skin and thymus fibroblasts, and FRCs. This analysis revealed the closest homology between colonic S4 cells with FRCs (Figure 4H). Since we identified corresponding clusters of fibroblast-like cells in the healthy and DSS datasets, we performed differential expression analysis between the identified subsets. The transcriptional responses to DSS challenge were dissimilar between mesenchymal subsets (Figure 4I).

### Divergence between Human and Murine Mesenchyme in Health and Colitis

**Figure 5:**
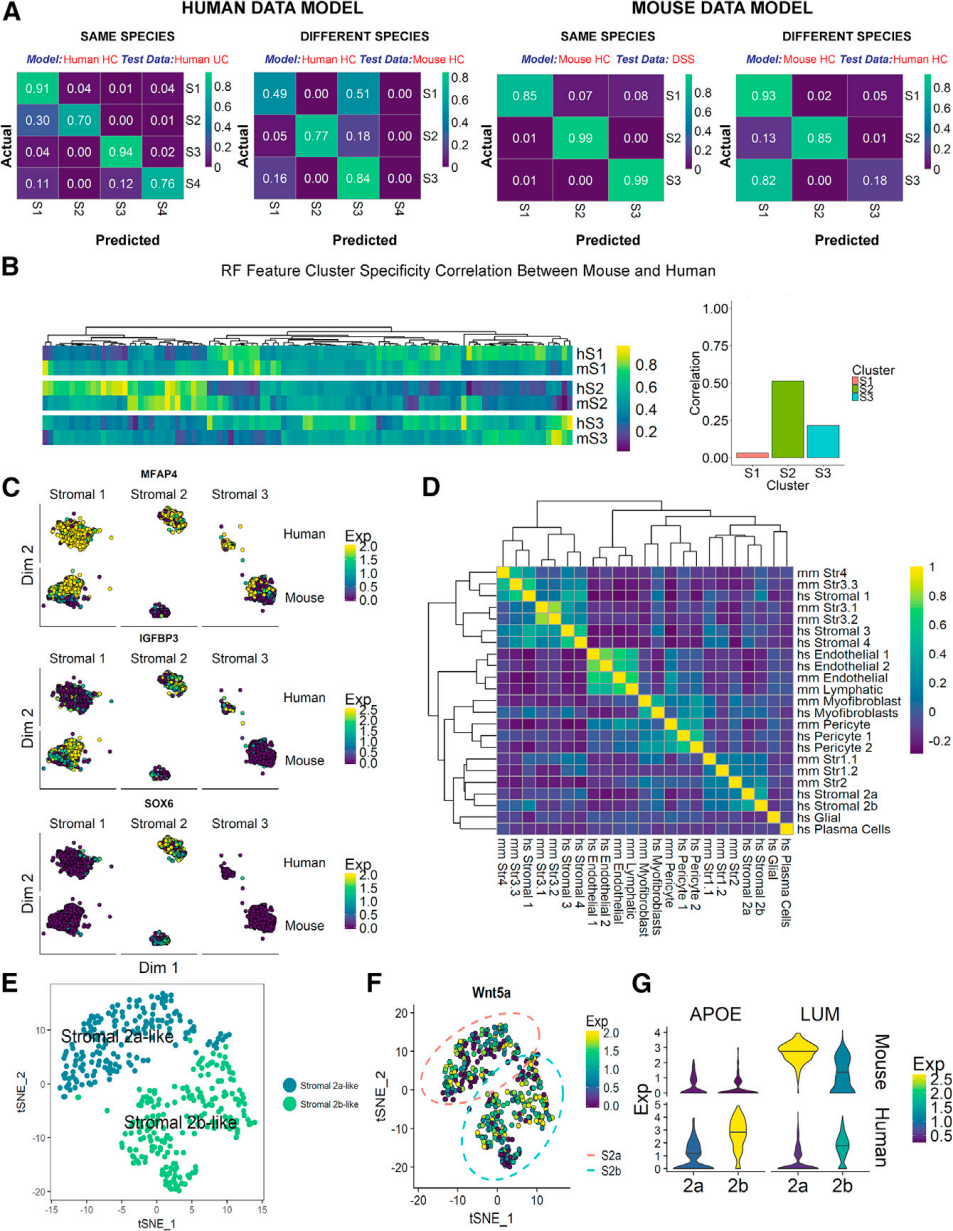
Comparing Murine and Human Colonic Mesenchymal Cells (A) Confusion matrices of human (left) and mouse (right) random forest models applied to independent datasets from the same species and different species show the proportion of real and model-predicted cell cluster identities for healthy control (HC), human UC, or mouse DSS. (B) Human HC model features scored for cluster specificity in human (hS1, hS2, hS3) and mouse (mS1, mS2, mS3) data. The heatmap shows increasingly positive cluster markers in yellow (>0.5) and increasingly negative cluster markers in purple (<0.5), and non-specific genes in green ( = 0.5). The bar plot shows the correlation between mouse and human marker specificity for each cluster. (C) Examples of features that drive the random forest results: MFAP4, IGFBP3, and SOX6. (D) Human and mouse cluster marker gene overlap correlation heatmap. (E) t-SNE plot visualizing sub-cluster analysis of S2 cells from healthy mouse scRNA-seq. Two distinct cell clusters, not previously detected, show similarities to human S2a and S2b counterparts. (F) Wnt5a expression by both S2a- and S2b-like mouse sub-clusters. (G) Violin plots show example S2 markers identified from human data that do not exhibit a conserved expression patterns in mouse S2 subtypes.

The DSS-induced mouse model of colitis is widely used to study mechanisms of IBD due to its simplicity and reproducibility, despite some key differences to the human disease. It is imperative to understand these differences both at the phenotypic and molecular level. Here, we used random forest models to compare the transcriptional profiles of human and mouse cells (Figure 5).

Initially, we selected cells from major healthy human stromal clusters (S1–S4) to train a four-class model and found it distinguished equivalent cell types in the human UC dataset with high sensitivity and specificity. (Figure 5A). The performance of the classifier on mouse data was notably worse for the S1 subset, as the model often misclassified mouse S1 cells as S3. This result may arise from similarities in S1 and S3 populations in the human samples. Remarkably, the majority of S2 and S3 mouse clusters were classified correctly, which suggests a degree of cross-species conservation in these cell populations. Next, we replicated this result training a reverse model with mouse expression data to classify the human data. Again, this model accurately identified most S2 cells, but it often classified the human S3 cluster as S1 (Figure 5A).

To investigate how gene expression drove the outcome of the model predictions, we examined how cluster specificity of the most informative genes selected by our human random forest model compared between human and mouse cell clusters. In agreement with the classification results, we found that gene specificity was most highly correlated between mouse and human S2 clusters, while S1 clusters showed little correlation, indicating less conserved gene expression patterns of this population (Figure 5B). For instance, healthy mouse S1 cells almost exclusively express *Igfbp3*, whereas human S1 cells do not and instead show greatest IGFBP3 expression in S2 and S3 cell populations (Figure 5C). *Mfap4* is a negative marker for mouse S2 cluster but shows ubiquitous expression across all human stromal clusters (Figure 5C). Nonetheless, some key marker genes showed good levels of conservation between mouse and human data, such as the key S2 transcription factor SOX6 (Figure 5C). When we analyzed the degree of overlap between human UC and mouse DSS cluster marker genes, we found that the human S1 cluster bore similarities to mouse S3 and S4 clusters, as well as bearing close similarity to human S3 and S4 subtypes, while other cell types showed higher levels of cluster marker preservation (e.g., endothelial cells segregate with endothelial cells, and all contractile types are together regardless of species) (Figure 5D). In the scRNA-seq data from healthy human patients, we readily detected two S2 subtypes; however, we did not observe this distinction in our initial mouse cluster analysis. To investigate this disparity, we isolated the S2 cluster from healthy mouse and performed unsupervised clustering analysis on this subset, which yielded two S2 sub-clusters (Figure 5E). We could match these subsets to their human 2a and 2b counterparts, owing to the conservation of key marker expression patterns, such as 2a-specific chemokine *Cxcl12*. We found higher expression of the membrane glycoprotein *Nrg1* in the 2b cell population in both human and mouse; however, we found expression of *Wnt5a* by both 2a-like and 2b-like populations in the mouse (Figure 5F), while it was localized to a S2b sub-cluster in human (Figure 1I). Similarly, a number of genes initially identified as S2 subtype-specific in humans did not segregate with S2a or 2b-like subsets in mouse (e.g., *Apoe*) or showed reversed cluster-specificity (e.g., *Lum*) (Figure 5G). It is difficult to determine whether these differences constitute a genuine phenotypic divergence between human and mouse or arose from technical, sampling, or environmental effects. Overall, these observations suggest crypt niche mesenchymal cells (S2) are broadly equivalent between mouse and human, whereas other mesenchymal subsets appear to lack homology, which reflects a lack of conservation for these specialized subsets.

### Decreased Mesenchymal Crypt Niche and Expanded Activated Mesenchymal Cell Markers in IBD

**Figure 6:**
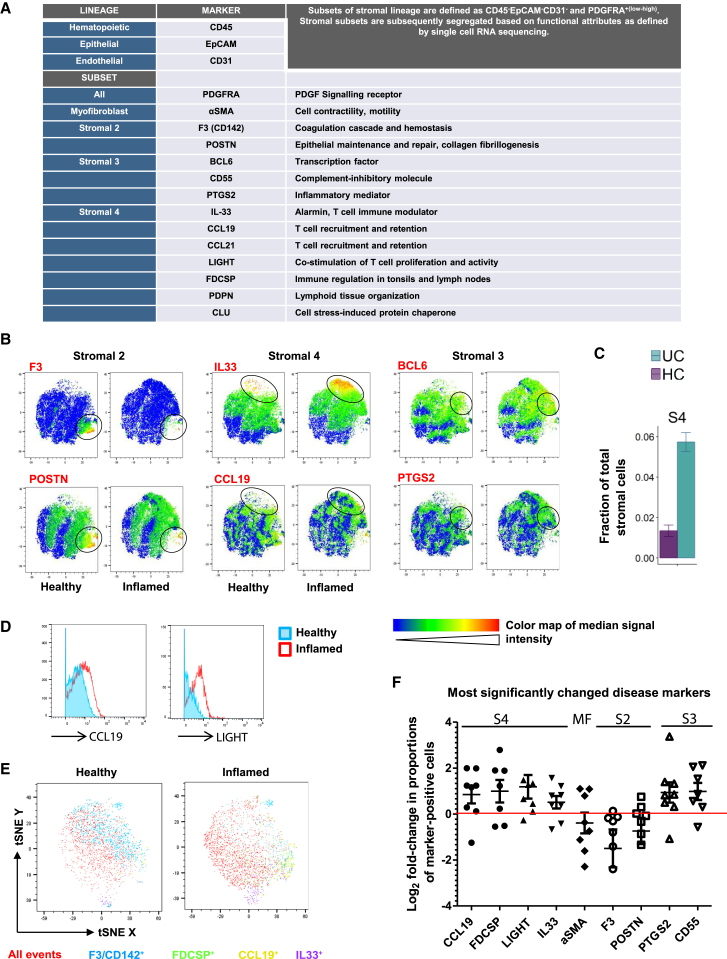
CYTOF Analysis of Key Mesenchymal Subset Markers Reveals Colitis-Associated Stromal Remodeling (A) CyTOF panel detected colonic mesenchymal populations. Stromal subsets are represented by indicated markers. (B) Heatmaps of selected markers on concatenated healthy and inflamed t-SNE plots representing key stromal subsets. Color maps by F3 (CD142), POSTN, IL-33, CCL19, BCL6, and PTGS2 shown. (C) Expansion of S4 in UC detected by scRNA-seq. (D) Histogram comparisons of CCL19 and TNFSF14 (LIGHT) levels in healthy versus inflamed colonic mesenchyme marks the emergence of S4. (E) t-SNE comparisons of healthy versus inflamed colonic mesenchyme. Clustering used the following parameters: F3/CD142, POSTN, PDGFRA, PDPN, BCL6, PTGS2, CD55, CCL19, CCL21, IL-33, LIGHT, CLU, FDCSP, and αSMA. Select markers representing S2 and S4 in healthy versus inflamed tissues shown. (F) Graphical summary of the most significantly changed markers in UC. Each dot represents one independent pair of healthy donor and patient samples. See also Figure S5.

**Figure S5:**
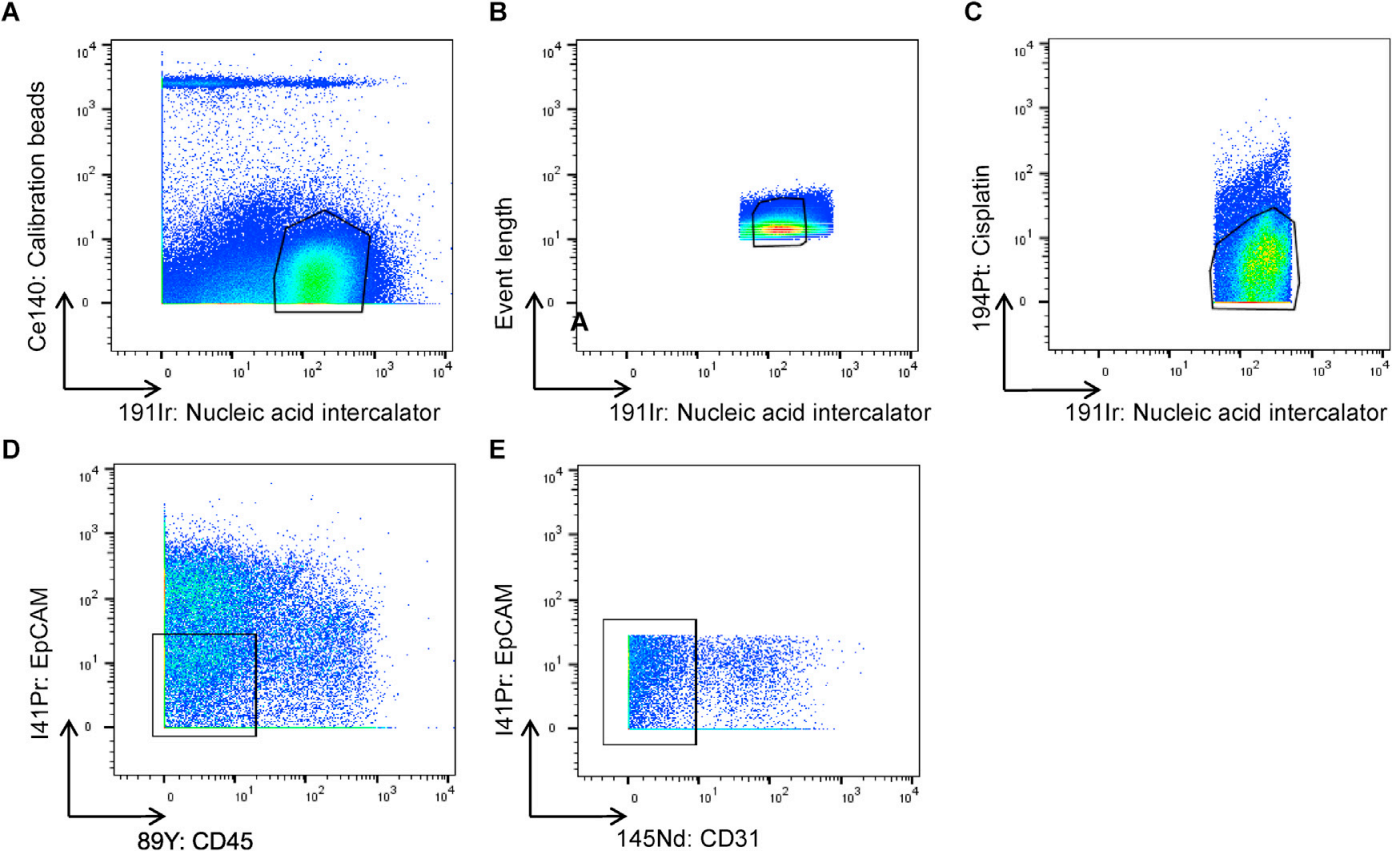
CyTOF Gating Strategies on Intestinal Stromal Cells from Human Colonic Biopsies, Related to Figure 6 (A–E) Gating was performed on cells (191Ir^+^140Ce^-^) to exclude calibration beads (A), followed by singlets (B), then live cells (C), and CD45^-^EpCAM^-^CD31^-^ events (D, E).

We then defined whether the extent of mesenchymal stromal remodeling found at the gene expression level also occurred at the protein level in IBD. We therefore developed a mass cytometry time of flight (CyTOF) panel designed to detect proteins whose expression segregates with the specific new mesenchymal subtypes we identified by scRNA-seq. We used CyTOF to circumvent tissue-associated auto-fluorescence and screened a variety of subset-associated proteins for their utility in CyTOF analysis, including cell-surface molecules, cytokines, and transcription factors (Figures 6A and S5). We observed several disease-associated changes in the UC stroma, exemplified by reduced S2 markers F3/CD142 and POSTN, increased BCL6 and PTGS2/COX-2 expression levels in S3, and markedly expanded S4. We found features of these pathogenic alterations reflected in t-distributed stochastic neighbor embedding (tSNE) analyses of the above markers in healthy versus inflamed colonic tissues (Figure 6B). These observations were consistent with scRNA-seq analyses, which also revealed a compositional shift toward a more S4-abundant phenotype in inflammation (Figure 6C). CyTOF examination of multiple pairs of healthy and inflamed colonic tissues demonstrated highly consistent upregulation of CCL19, FDCSP, TNFSF14 (LIGHT), and IL-33 in disease (Figures 6D–6F), reflecting the emergence of a strong S4 signature. Other significantly changed subset-associated markers represent subsets 2–4 (Figure 6F), while the myofibroblast subset remained relatively unchanged in inflammation. Collectively, these data demonstrate CyTOF can monitor pathogenic colonic mesenchymal behavior in inflamed tissues and capture changes correlative of clinical disease activity in IBD.

### Functional Attributes of Crypt Niche and Disease-Associated Colonic Mesenchymal Cells in Health and IBD

**Figure 7:**
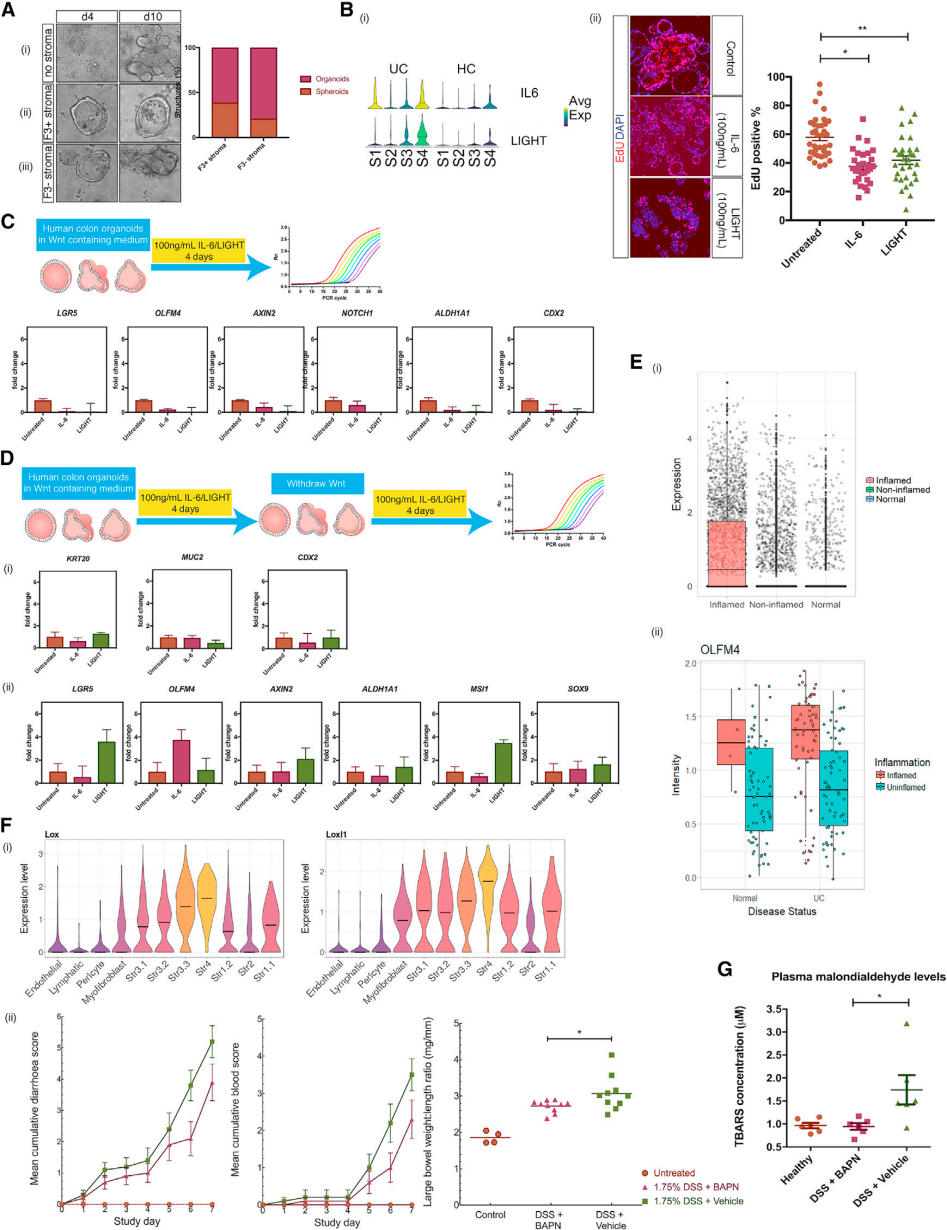
Functional Attributes of Crypt Niche and IBD-Associated Mesenchymal Cells (A) Epithelial characterization after *in vitro* co-culture with and without S2. S2 was isolated by fluorescence-activated cell sorting (FACS) for F3 (CD142). Crypts with (ii) and without (iii) F3^+^ stromal cells grown in culture containing Rspo1 and assessed for up to 10 days of culture. Representative images from day 4 and day 10 are shown. (i) Normal growth of human colon organoids without any stromal cells. Bar graph shows quantification of organoid complexity during the course of co-culture. (B) (i) Violin plots from the scRNA-seq data showing IL-6 and TNFSF14 (LIGHT) upregulated by S4. (ii) Human colon organoids were treated with 100 ng/mL of either IL-6 or LIGHT. Confocal immunofluorescence images show EdU-labeled nuclei (red) and total nuclei stained with DAPI (blue). Epithelial proliferative capacity was assessed by quantification of the total numbers of EdU positive nuclei and DAPI-stained nuclei to calculate the fraction of proliferating cells in a section of interest. For each experiment, 15 random fields were quantified for each treatment. n = 3 independent experiments. ^∗∗^p < 0.0001, ^∗^p < 0.001 Mann-Whitney U test. (C) Real-time qPCR measured stem cell markers (*LGR5*, *OLFM4*, *AXIN2*, *NOTCH1*, and *ALDH1A1*) and *CDX2* gene expression after treatment of human colon organoids with IL-6 or LIGHT for 4 days in the presence of Wnt containing medium. (D) Real-time qPCR measured stem cell marker (*LGR5*, *OLFM4*, *AXIN2*, *ALDH1A1*, *MSI1*, and *SOX9*) and differentiation marker (*KRT20*, *MUC2*, and *CDX2*) gene expression after treatment of human colon organoids with IL-6 or LIGHT for 4 days in the presence of Wnt containing medium, with subsequent Wnt withdrawal and treatment with IL-6 and LIGHT for another 4 days. (E) *OLFM4* gene expression from scRNA-seq of over 11,175 single cells isolated from healthy, non-inflamed and inflamed colonic biopsies (i), and gene expression from bulk RNA of inflamed and non-inflamed mucosa of IBD patients compared to healthy control samples. (F) (i) Violin plots of relative gene expression of *Lox* and *Loxl1* in DSS-induced colitis. (ii) Cumulative diarrhea score, blood score, and large bowel weight to length ratio of vehicle-only Ctrls versus BAPN-treated animals. (G) Lipid peroxidation measured by malondialdehyde (MDA) plasma levels of vehicle-only and BAPN-treated animals. Error bars represent the SEM.

The localization of S2 cells close to the base of the colonic crypt (Figure 1E) and the factors they secrete (Figures 1D and 1E) suggest a role to support intestinal epithelial stem cell function. To test this, we used a “mini-gut” culture system (Sato et al., 2011) that allows the growth of human colonic crypts into organoids. In the absence of any stromal cells but presence of exogenous growth factors, human colonic crypts containing intestinal epithelial stem cells spontaneously formed self-organizing structures and differentiated into multi-fingered organoids after 10 days in culture (Figure 7Ai). Adding F3^+^ stromal cells from healthy human colon led to the formation of spherical structures termed spheroids with very low levels of organoid budding over 10 days (Figure 7Aii). In contrast, crypts cultured with F3^–^ stromal cells changed from a spheroid morphology into budding organoids over the same time course (Figure 7Aiii). These results reflect events in stromal cell-free culture, where removal of Wnt and Nicotinamid from the organoid media caused sphere-like organoids to bud (Schwank et al., 2013). Our data suggest that S2 cells promote colonic epithelial stem cell maintenance.

Next, we investigated the effects of UC-associated S4 cells on the epithelium. We measured the effects of two S4 secreted factors, IL-6 and TNFSF14 (LIGHT), (Figure 7Bi) on epithelial proliferative capacity using immunofluorescence and confocal microscopy. As observed in Figure 7Bii), we found stimulation with both IL-6 and LIGHT led to a reduction in DNA replication using a short pulse of ethynyldeoxyuridine (EdU) as a measure of S phase cells.

We also tested the effect of these secreted factors on expression of intestinal stem cell and Wnt-responsive genes by real-time qPCR. Organoids treated with IL-6 or LIGHT for 4 days showed a marked decrease in expression of *LGR5*, *OLFM4*, *AXIN2*, *ALDHA1*, *CDX2*, and *NOTCH1* (Figure 7C). To replicate the conditions of S2 depletion and S4 expansion in UC (Figures 6E and 6F), we performed the same experiment as above with IL-6 or LIGHT stimulation for 4 days following WNT withdrawal from the organoid medium. We then stimulated cells with IL-6 or LIGHT for another 4 days and quantified their effects by real-time qPCR. Interestingly, after withdrawal of WNT, LIGHT stimulation increased expression of typical stem cell markers (Figure 7D), such as *LGR5*, *OLFM4*, and *AXIN2*. We also found upregulated *SOX9* and *MSI1*, considered damage-responsive “reserve” stem cell markers. However, other reserve stem cell markers (Barker, 2014), such as *LRIG1*, *HOPX*, *BMI1*, *PROM1*, *EPHB2*, and *KLF4*, showed little or no change compared to untreated epithelial organoids (data not shown). IL-6 stimulation also induced an approximate 5-fold change in *OLFM4* gene expression. We found no changes in expression of various differentiation markers in every condition. Interestingly, scRNA-seq data generated in our lab from over 11,175 epithelial cells comparing healthy and UC patients showed a marked increase in *OLFM4* expression (Figure 7Ei) in the stem cells from inflamed tissues. We confirmed this observation by querying the GEO database (Edgar et al., 2002). This analysis revealed *OLFM4* upregulation in inflamed biopsies of UC patients compared to paired biopsies from uninflamed regions (Figure 7Eii) from a genome-wide expression study comparing biopsies from 67 patients with UC and 31 control subjects (23 normal and 8 patients with inflamed non-IBD biopsies) (GEO accession GSE11223; Noble et al., 2008). Taken together, our data suggest each sub-group of stromal cells has a defined role to maintain and regenerate the intestinal epithelium during health and disease.

### Pathogenic Stromal Activity Exacerbates Colitis through Redox Imbalances

The Lox family of lysyl oxidase enzymes catalyze covalent crosslinking of collagen and elastin, generating hydrogen peroxide as a by-product (Csiszar, 2001) that elicits both tissue-local and systemic redox disturbances that perpetuate inflammation. In S4 cells from DSS colitis, *Lox* and *Loxl1* are induced with high mesenchymal-specific expression (Figure 7Fi). Since oxidant stressors are inflammatory chemoattractants and factors in IBD pathogenesis, we hypothesized that blockade of Lox enzymes may decrease colitis severity. We administered the Lox/Loxl1 inhibitor β-aminopropionitrile (BAPN) to colitic animals. This treatment improved multiple disease parameters, including diarrhea score, cumulative blood score, and the colon weight to length ratio (Figure 7Fii). To assess oxidative damage, we measured malondialdehyde (MDA) levels as an indicator of lipid peroxidation in the plasma of these animals. Inhibiting Lox enzymes completely normalized plasma MDA levels to those of healthy controls (Figure 7G), indicating Lox enzyme activities are the predominant source of systemic oxidative stress in DSS-induced colitis. Therefore, IBD-associated S4 is equipped to elicit redox imbalances to sustain inflammation and to induce proinflammatory factors.

## Discussion

**Figure S6:**
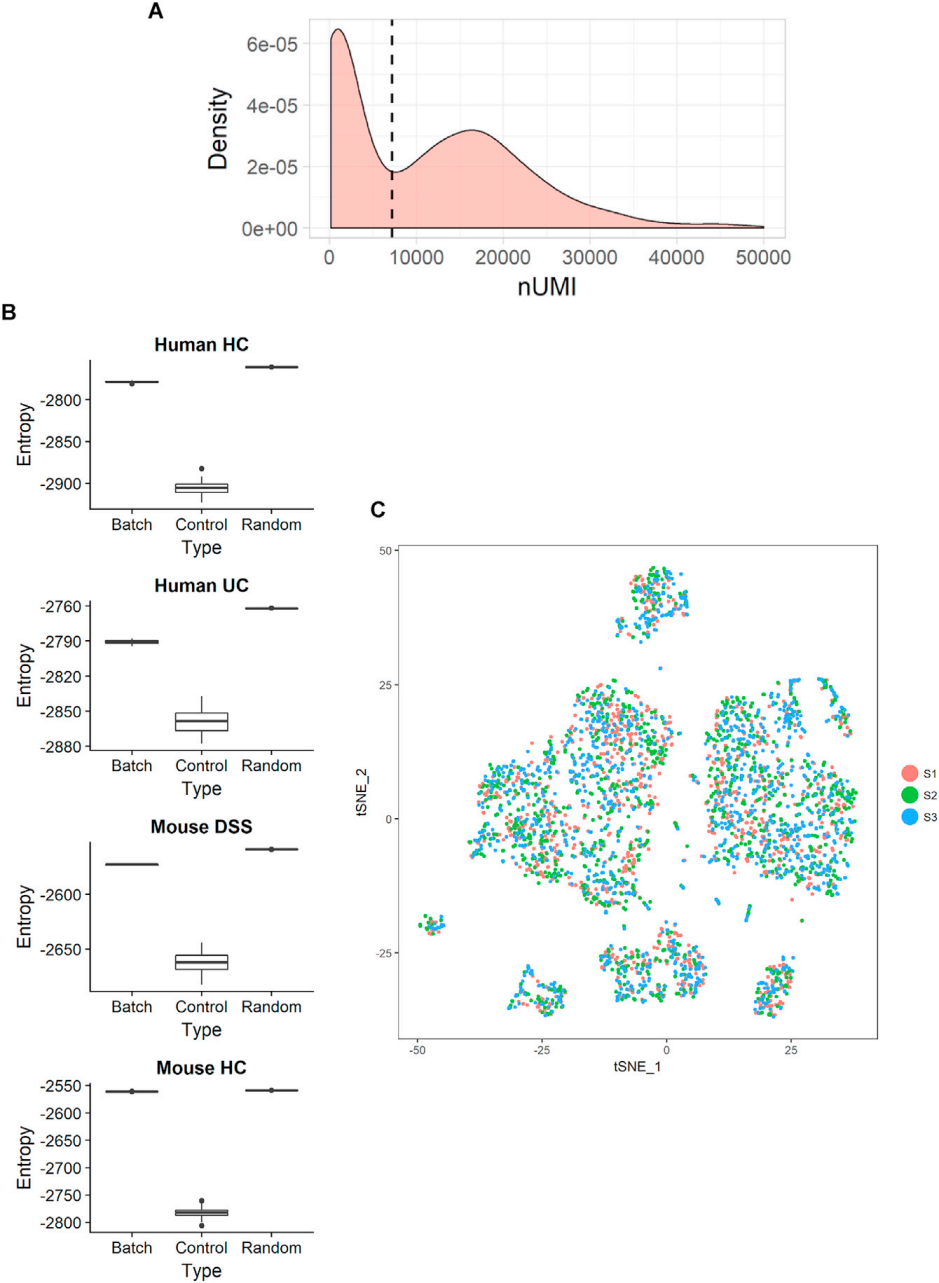
Computational Analysis and Batch Effect Assessment, Related to Quantification and Statistical Analysis (A) Identification of cellular barcodes in 10x data was selected as the first local minima across individual samples. Example distribution density and local minimum (dashed line) are shown. (B) Batch effects in the 10x scRNA-seq data. Boxplots show the entropy of batch mixing for each dataset (Batch), compared to a negative (Random) and positive (Control) controls. For each set of data, entropy of batch mixing was computed as in Haghverdi et al., 2018. As negative controls (no batch effect), random batch labels were assigned to each cell. As a set of positive batch controls (each cluster is driven entirely by batch effect), cluster labels were used. In each dataset, the entropy of mixing for the batch effects approaches that of negative control. (C) tSNE plot visualizing the batch distribution in healthy mouse 10x data, corresponding to S2B bottom panel.

Intestinal mesenchymal cells direct a complex network of cross-talk between immune, endothelial, and epithelial compartments, balancing tissue structural integrity and mucosal tolerance to bacterial and environmental antigens. Here, we undertook a single-cell census to define the extent of cellular heterogeneity within the colonic mesenchyme in mouse and man in health and colitis, with highly consistent results across all samples tested (Figure S6). We identified populations of established cells, such as myofibroblasts and pericytes, and four additional distinct populations of fibroblast-like cells.

We identified a colonic crypt niche mesenchymal S2 population, which expressed F3/CD142 and the transcription factor SOX6 located in direct proximity to epithelial cells (Figure 1). S2 was enriched for WNTs essential for stem cell self-renewal. In contrast to the small intestine, the colonic crypt does not harbor Paneth cells and relies on non-epithelial sources for Wnt ligands (San Roman et al., 2014). Two recent murine studies deleted key proteins required for Wnt secretion in Foxl1- and Gli1-producing intestinal stromal cells, respectively, which led to crypt collapse and further supports S2 classification as a mesenchymal niche cell (Degirmenci et al., 2018, Shoshkes-Carmel et al., 2018). We observed remodeling of S2 in IBD, likely contributing to epithelial barrier breakdown, which is a hallmark of this disease (Figure 6). S2 consisted of two subpopulations (2a and 2b) and the role of each in crypt maintenance, inflammation, and cancer will be an important subject for further investigation.

In colitis, we observed the emergence of S4, which uniquely gained lymph node FRC-like features (Figure 4H). We found S4-expressed Lox and Loxl1 blockade attenuated DSS colitis and reduced circulating markers of oxidative stress (Figures 7F and 7G). IL-6 and TNFSF14 restricted colonic epithelial cell proliferation and induced expression of stemness genes, such as *Lgr5* (Figures 7B–7E). This may reflect recruitment of normally quiescent epithelial “label-retaining cells” (LRCs), that are recalled to the stem cell compartment following inflammation mediated injury (Buczacki et al., 2013). Overall, we demonstrate stromal remodeling in IBD is functionally divergent in a subset-specific manner, where normal repair and regeneration responses mediated by crypt niche S2 are compromised, while continuous production of pro-inflammatory S4 factors prevent the resolution phase of a wound-healing response (Figure 6).

Our study will enable future generations of Cre-expressing reporter and fate-mapping mouse lines to illuminate lineage relationships and functions of novel mesenchymal subtypes *in vivo*. Rinkevich et al. (2012) identified a mesothelial precursor lineage for colonic stromal cells. We identified *Wt1* as a mesothelial marker segregating within murine S3 (Figure 3G), a possible progenitor population suggested by trajectory analysis (Figure 4A). S3 also demonstrated increased mitotic activity (Figure 4G) in support of this hypothesis. Understanding the pathways underlying trans-differentiation will pinpoint mechanisms to enhance specific functional features of these cells and restore tissue homeostasis in diseases like IBD.

Up to 40% of IBD patients fail to respond to conventional immunotherapies. Our work demonstrates the utility of single-cell approaches to define common and divergent features of inflammatory diseases among species. This knowledge will better inform the design of updated models for drug development. Reducing complex scRNA-seq data to simple immune monitoring panels, such as the CyTOF panel generated in this work, will enhance stratification and immune monitoring of existing and new therapies in IBD.

## STAR★Methods

### Key Resources Table

**Table:** 

REAGENT or RESOURCE	SOURCE	IDENTIFIER
**Antibodies**		

Mouse monoclonal anti-human CD45	BioLegend	Cat# 304023; RRID: AB_493760
Mouse monoclonal anti-human CD31	BioLegend	Cat#303121; RRID: AB_2562148
Donkey polyclonal anti-rabbit IgG	BioLegend	Cat#406410; RRID: AB_10897810
Mouse monoclonal anti-human CD24	BioLegend	Cat#311135; RRID: AB_2566578
Goat polyclonal anti-mouse IgG	BioLegend	Cat#405308; RRID: AB_315011
Rat monoclonal anti-human PDPN	BioLegend	Cat#337011; RRID: AB_2561308
Mouse monoclonal anti-human EpCAM	BioLegend	Cat#324205; RRID: AB_756079
Mouse monoclonal anti-human CD74	eBioscience	Cat#11-0748-41; RRID: AB_2043845
Recombinant human IgG1 anti-human fibroblast antigen	Miltenyi	Cat#130-100-139; RRID: AB_2651744
Rabbit polyclonal anti-human FDCSP	abcam	Cat#ab121420; RRID: AB_11127721
Mouse monoclonal anti-human SOX6	abcam	Cat#ab84880; RRID: AB_1861338
Mouse monoclonal anti-human ZEB1	Atlas Antibodies	Cat#AMAb90510; RRID: AB_2665569
Rabbit polyclonal anti-human ZEB2	Atlas Antibodies	Cat#HPA003456; RRID: AB_10603840
Goat polyclonal anti-human LIGHT/TNFSF14	R and D Systems	Cat#AF664; RRID: AB_355512
Goat polyclonal anti-human/mouse COX-2	Bio-Techne	Cat#AF4198; RRID: AB_2229909
Goat polyclonal anti-human Coagulation Factor III/Tissue Factor	Bio-Techne	Cat#AF2339; RRID: AB_442150
Mouse monoclonal anti-human SOX6	Bio-Techne	Cat#MAB7759, RRID: AB_2737259
Goat polyclonal anti-human/mouse Bcl-6	Bio-Techne	Cat#AF5046; RRID: AB_2063454
Mouse monoclonal anti-human CD55/DAF	Bio-Techne	Cat#MAB20091; RRID: AB_2075960
Goat polyclonal anti-human CCL21/6Ckine	Bio-Techne	Cat#AF366; RRID: AB_355327
Mouse monoclonal anti-human CCL19/MIP-3 beta	Bio-Techne	Cat#MAB361; RRID: AB_2071417
Sheep polyclonal anti-human Podoplanin	Bio-Techne	Cat#AF3670; RRID: AB_2162070
Mouse monoclonal anti-human ZEB1	Bio-Techne	Cat#MAB6708; RRID: AB_10972647
Mouse monoclonal anti-human ZEB2/SIP1	Bio-Techne	Cat#MAB73782; RRID: AB_2737260
Mouse monoclonal anti-human IL-33 (6H617)	Bio-Techne	Cat#NBP2-27333; RRID: AB_2737261
Mouse monoclonal anti-human/mouse/rat alpha -Smooth Muscle Actin	Bio-Techne	Cat#MAB1420; RRID: AB_262054
Goat polyclonal anti-human PDGF R alpha	Bio-Techne	Cat#AF-307-NA; RRID: AB_354459
Mouse monoclonal anti-human Clusterin	Bio-Techne	Cat#MAB2937; RRID: AB_2229755
Rat anti-human/Mouse Periostin/OSF-2 Antibody	Bio-Techne	Cat#MAB3548; RRID: AB_2252599
Mouse monoclonal anti-F3	Atlas Antibodies	Cat#AMAb91235; RRID:AB_2665858
Rabbit polyclonal anti-SMAD7	Atlas Antibodies	Cat#HPA028897; RRID: AB_10600811
Rat monoclonal anti-CD45R (RA3-6B2)	Abcam	Cat#ab64100; RRID: AB_1140036
Mouse monoclonal anti-CD45 (2B11 + PD7/26)	Agilent (Dako)	Cat#M0701; RRID: AB_2314143
Mouse monoclonal anti-human CD90	BioLegend	Cat#328107; RRID: AB_893438
Anti-Human CD326/EpCAM (9C4)-141Pr	Fluidigm	Cat#3141006B; RRID: AB_2687653
Anti-Human CD45 (HI30)-Y89	Fluidigm	Cat#3089003B; RRID: AB_2661851
Anti-Human CD31/PECAM-1 (WM59)-145Nd	Fluidigm	Cat#3145004B; RRID: AB_2737262
Maxpar X8 Antibody Labeling Kit, 144Nd	Fluidigm	Cat#201144B
Maxpar X8 Antibody Labeling Kit, 146Nd	Fluidigm	Cat#201146B
Maxpar X8 Antibody Labeling Kit, 150Nd	Fluidigm	Cat#201150B
Maxpar X8 Antibody Labeling Kit, 151Eu	Fluidigm	Cat#201151B
Maxpar X8 Antibody Labeling Kit, 152Sm	Fluidigm	Cat#201152A
Maxpar X8 Antibody Labeling Kit, 154Sm	Fluidigm	Cat#201154B
Maxpar X8 Antibody Labeling Kit, 159Tb	Fluidigm	Cat#201159B
Maxpar X8 Antibody Labeling Kit, 160Gd	Fluidigm	Cat#201160B
Maxpar X8 Antibody Labeling Kit, 162Dy	Fluidigm	Cat#201162B
Maxpar X8 Antibody Labeling Kit, 164Dy	Fluidigm	Cat#201164B
Maxpar X8 Antibody Labeling Kit, 166Er	Fluidigm	Cat#201166B
Maxpar X8 Antibody Labeling Kit, 167Er	Fluidigm	Cat#201167B
Maxpar X8 Antibody Labeling Kit, 168Er	Fluidigm	Cat#201168B
Maxpar X8 Antibody Labeling Kit, 169Tm	Fluidigm	Cat#201169B
Maxpar X8 Antibody Labeling Kit, 172Yb	Fluidigm	Cat#201172B
Maxpar X8 Antibody Labeling Kit, 173Yb	Fluidigm	Cat#201173B
Maxpar X8 Antibody Labeling Kit, 175Lu	Fluidigm	Cat#201175B
Maxpar X8 Antibody Labeling Kit, 176Yb	Fluidigm	Cat#201176B

**Biological Samples**		

Human intestinal tissue biopsies	University of Oxford Translational Gastroenterology Unit	https://www.expmedndm.ox.ac.uk/tgu/tgu-biobank-ibd-cohort
Murine colon	Epistem	This study

**Chemicals, Peptides, and Recombinant Proteins**		

4′,6-Diamidine-2′-phenylindole dihydrochloride	Sigma Aldrich	Cat#10236276001
Dispase II, powder	ThermoFisher Scientific	Cat#17105041
Matrigel Basement Membrane Matrix Growth Factor Reduced (GFR) Phenol Red-Free LDEV-Free	Corning	Cat#356231
SB 431542	R&D Systems	Cat#1614/10
Y-27632 dihydrochloride	R&D Systems	Cat#1254/10
CTS GlutaMAX-I Supplement	ThermoFisher Scientific	Cat#A1286001
N-2 Supplement (100X)	ThermoFisher Scientific	Cat#17502048
B-27 Supplement (50X), serum free	ThermoFisher Scientific	Cat#17504044
NICOTINAMIDE	Sigma Aldrich	Cat#N0636
Recombinant Human EGF	PeproTech	Cat#AF-100
Recombinant Human IL-6	R&D Systems	Cat#206-IL-010
Recombinant Human LIGHT/TNFSF14	R&D Systems	Cat#664-LI-025
A 83-01	R&D Systems	Cat#2939/10
[LEU15]-Gastrin I HUMAN	Sigma Aldrich	Cat#G9145
Prostaglandin E2	Sigma Aldrich	Cat#P0409
N-Acetylcysteine	Sigma Aldrich	Cat#A9165
Recombinant Human R-Spondin-1	PeproTech	Cat#120-38
Recombinant Murine Noggin	PeproTech	Cat#250-38
2.5% Normal Goat Serum	Vector Laboratories	Cat#S-1012
ImmPRESS HRP Anti-Rabbit IgG (Peroxidase) Polymer Detection Kit, made in Goat	Vector Laboratories	Cat#MP-7451; RRID: AB_2631198
ImmPRESS HRP Anti-Mouse IgG (Peroxidase) Polymer Detection Kit, made in Goat	Vector Laboratories	Cat#MP-7452
ImmPRESS-AP Anti-Rabbit IgG (alkaline phosphatase) Polymer Detection Kit	Vector Laboratories	Cat#MP-5401; RRID: AB_2336536
ImmPRESS-AP Anti-Mouse IgG (alkaline phosphatase) Polymer Detection Kit	Vector Laboratories	Cat#MP-5402; RRID: AB_2336535
ImmPACT DAB Peroxidase (HRP) Substrate	Vector Laboratories	Cat#SK-4105; RRID: AB_2336520
Vector Blue Alkaline Phosphatase (Blue AP) Substrate Kit	Vector Laboratories	Cat#SK-5300; RRID: AB_2336837
ImmPACT VIP Peroxidase (HRP) Substrate	Vector Laboratories	Cat#SK-4605; RRID: AB_2336525
Dako Liquid Permanent Red	Agilent (Dako)	Cat#K0640
Lab Vision Ultra V Block	Fisher scientific	Cat#12583158
ACK Lysing Buffer	Thermo Fisher	Cat#A1049201
Bovine Serum Albumin	Sigma	Cat#A1933
CellTracker Green CMFDA Dye	Thermo Fisher	Cat#C7025
CellTracker Orange CMRA Dye	Thermo Fisher	Cat#C34551
Collagenase VIII	Sigma Aldrich	Cat#C2139
DMEM	Sigma	Cat#D5671
Dextran Sodium Sulfate (DSS)	MP Biomedicals	Cat#M9147
UltraPure 0.5M EDTA, pH 8.0	Thermo Fisher	Cat#15575020
Foetal Bovine Serum	Sigma	Cat#F9665
HBSS, no calcium, no magnesium	Thermo Fisher	Cat#14170112
HEPES solution	Sigma	Cat#H0887
LIVE/DEAD Fixable Dead Cell Stain	Thermo Fisher	Cat#L10120
NucBlue Live Reagent	Thermo Fisher	Cat#R37605
Percoll	GE Healthcare	Cat#17-0891-01
Propidium Iodide Solution	BioLegend	Cat#421301

**Critical Commercial Assays**		

Foxp3 / Transcription Factor Staining Buffer Set	eBioscience	Cat#00-5523-00
Cytofix/Cytoperm	BD Biosciences	Cat#554714
Lightning-Link APC Antibody Labeling Kit	Novus Biologicals	Cat#705-0030
Lightning-Link R-PE Antibody Labeling Kit	Novus Biologicals	Cat#703-0030
Click-iT EdU Alexa Fluor 647 Imaging Kit	ThermoFisher Scientific	Cat#C10340
RNeasy Mini Kit (250)	QIAGEN	Cat#74106
iScript cDNA Synthesis Kit	Bio-Rad	Cat#1708891
RNAscope 2.5 HD Reagent Kit - BROWN	ACD Europe SRL	Cat#322300
Maxpar Nuclear Antigen Staining Buffer Set	Fluidigm	Cat#201063
Maxpar Cell Staining Buffer	Fluidigm	Cat#201068
CD45 Microbeads, human	Miltenyi Biotec	Cat#130-045-801
CD45 MicroBeads, mouse	Miltenyi Biotec	Cat#130-052-301
CD235a MicroBeads, human	Miltenyi Biotec	Cat#130-050-501
CD326 (EpCAM) MicroBeads, human	Miltenyi Biotec	Cat#130-061-101
CD326 (EpCAM) MicroBeads, mouse	Miltenyi Biotec	Cat#130-105-958
Chromium Single Cell 3′ Library & Gel Bead Kit v2	10X Genomics	Cat#120237
LD Columns	Miltenyi Biotec	Cat#130-042-901
Nextera XT DNA Library Preparation Kit	Illumina	Cat#FC-131-1096
SMARTer Ultra Low RNA Kit	Clontech	Cat#634832
Umbilical Cord Dissociation Kit human	Miltenyi Biotec	Cat#130-105-737
Chromium Single Cell 3′ Library & Gel Bead Kit v2	10X Genomics	Cat#PN-120237
Cell-ID Cisplatin-194Pt	Fluidigm	Cat#201194
EQ Four Element Calibration Beads	Fluidigm	Cat#201078
Cell-ID Intercalator-Ir	Fluidigm	Cat#201192A

**Deposited Data**		

Single-cell RNaseq data	This study	GEO: GSE95459
Single-cell RNaseq data	This study	GEO: GSE114374

**Experimental Models: Cell Lines**		

L Wnt-3A (male)	ATCC	Cat#CRL-2647; RRID:CVCL_0635
RAW 264.7 (male)	ATCC	Cat#TIB71; RRID:CVCL_0493

**Experimental Models: Organisms/Strains**		

C57BL/6 male mice	Envigo Laboratories	RRID:IMSR_JAX:000664

**Oligonucleotides**		

*LGR5*	ThermoFisher Scientific	Cat#Hs00969422_m1
*OLFM4*	ThermoFisher Scientific	Cat#Hs00610344_m1
*AXIN2*	ThermoFisher Scientific	Cat#Hs00610344_m1
*NOTCH1*	ThermoFisher Scientific	Cat#Hs01062014_m1
*ALDH1A1*	ThermoFisher Scientific	Cat#Hs00946916_m1
*SOX9*	ThermoFisher Scientific	Cat#Hs00165814_m1
*MSI1*	ThermoFisher Scientific	Cat#Hs01045894_m1
*KRT20*	ThermoFisher Scientific	Cat#Hs00300643_m1
*MUC2*	ThermoFisher Scientific	Cat#Hs03005103_g1
*CDX2*	ThermoFisher Scientific	Cat#Hs01078080_m1
*HPRT1*	ThermoFisher Scientific	Cat#Hs02800695_m1
*GAPDH*	ThermoFisher Scientific	Cat#Hs02786624_g1
RNAscope Probe - Hs-ADAMDEC1	ACD Europe SRL	Cat#478471
RNAscope Probe - Hs-DCN	ACD Europe SRL	Cat#589521
RNAscope Probe - Hs-SLIT2	ACD Europe SRL	Cat#466221
RNAscope Probe - Hs-CXCL12	ACD Europe SRL	Cat#422991
RNAscope Probe - Hs-F3	ACD Europe SRL	Cat#407611
RNAscope Probe - Hs-HSD17B2	ACD Europe SRL	Cat#467271
RNAscope Probe - Hs-POSTN	ACD Europe SRL	Cat#409181
RNAscope Probe - Hs-FRZB	ACD Europe SRL	Cat#412391
RNAscope Probe - Hs-WNT5A	ACD Europe SRL	Cat#604921
RNAscope Probe - Hs-WNT5B	ACD Europe SRL	Cat#423041
RNAscope Probe - Hs-BMP2	ACD Europe SRL	Cat#430641
RNAscope Probe - Hs-BMP5	ACD Europe SRL	Cat#472461
RNAscope Probe - Hs-FDCSP	ACD Europe SRL	Cat#444231

**Software and Algorithms**		

ComBat sva Version 3.28.0	R Bioconductor	RRID: SCR_012836; https://bioconductor.org/packages/release/bioc/html/sva.html
CellRanger Version 2.0 Version 2.1.1	10X Genomics	https://support.10xgenomics.com/single-cell-gene-expression/software/downloads/latest
Seurat Version 1.4.0.16 Version 2.3.0	R Bioconductor	RRID: SCR_016341; https://www.satijalab.org/seurat
Scran Version 1.2.0 Version 1.6.9	R Bioconductor	https://github.com/MarioniLab/scran
Caret Version 6.0-80	CRAN	https://cran.r-project.org/web/packages/caret/index.html
DMrW Version 0.4.1	CRAN	https://cran.r-project.org/web/packages/DMwR/index.html
Biomart Version 92	Ensembl	RRID: SCR_002344; https://www.ensembl.org/biomart/martview/
pROC Version 1.12.1	CRAN	https://cran.r-project.org/web/packages/pROC/index.html
clusterProfiler Version 3.8.1	R Bioconductor	https://github.com/GuangchuangYu/clusterProfiler
Destiny Version 2.10.2	R Bioconductor	https://www.helmholtz-muenchen.de/icb/destiny
FastQC (v0.10.1)	Babraham Bioinformatics	RRID: SCR_014583; http://www.bioinformatics.babraham.ac.uk/projects/fastqc/
FeatureCounts (v1.5.0)	Source Forge	RRID: SCR_012919; http://subread.sourceforge.net
HISAT (v2.0.3b)	Kim et al., 2015	RRID: SCR_015530; https://ccb.jhu.edu/software/hisat/index.shtml
MultiQC (v0.7)	Ewels et al., 2016	RRID: SCR_014982; http://multiqc.info
NMF (v0.20.6)	CRAN	https://cran.r-project.org/web/packages/NMF/index.html
Picard Tools (v2.3.0)	Github	RRID: SCR_006525; http://broadinstitute.github.io/picard/
RUVSeq (v1.8.0)	Github	RRID: SCR_006263; https://github.com/drisso/RUVSeq
scater (v1.2.0)	Github	RRID: SCR_015954; https://github.com/davismcc/scater
Skewer	Github	RRID: SCR_001151; https://github.com/relipmoc/skewer
RTsne	CRAN	RRID: SCR_016342; https://cran.r-project.org/web/packages/Rtsne/index.html
WGCNA (v1.51)	CRAN	RRID: SCR_003302; https://cran.r-project.org/web/packages/WGCNA/index.html
RandomForest Version 4.6	CRAN	RRID: SCR_015718; https://cran.r-project.org/web/packages/randomForest/index.html

**Other**		

ACD HybEZ Hybridization System (220v)	ACD Europe SRL	Cat#310013
Hg38 reference genome	UCSC	http://hgdownload.cse.ucsc.edu/goldenPath/hg38/bigZips/
Mm10 reference genome	UCSC	http://hgdownload.cse.ucsc.edu/goldenPath/mm10/bigZips/

### Contact for Reagent and Resource Sharing

Further information and requests for resources and reagents should be directed to and will be fulfilled by the Lead Contact, Alison Simmons (alison.simmons@imm.ox.ac.uk).

### Experimental Model and Subject Details

#### Human studies

Colonic biopsy samples were collected from patients attending for clinically indicated endoscopy procedures at Oxford University Hospitals NHS Foundation Trust (OUHFT) following written informed consent. Inclusion criteria were male or female adults aged 18–90 years, mental capacity to give informed consent for study participation, and proficient in understanding written and verbal English. Exclusion criteria were the presence of a defined genetic syndrome predisposing to colorectal cancer, a family history of colorectal cancer defined as high or moderate risk, any contraindication to endoscopic forceps biopsy, or significant co-morbidity. Samples from 10 subjects were used in scRNA-seq experiments (Table S1). NHS National Research Ethics Service (NRES) research ethics committee (REC) references for the study include 14/YH/1116; GI 16/YH/0247 and IBD 09/H1204/30.

#### Animal studies

Murine tissue was generated in a DSS challenge model conducted by Epistem® Ltd. All procedures were certified according to the UK Home Office Animals (Scientific Procedures) Act 1986 (project license P9B86E6FD). C57BL/6 (*Helicobacter pylori*-free, murine norovirus-free) male mice (Envigo Laboratories, UK) were used in the study. Animals were 8–10 weeks old on supply and used at 10–12 weeks of age. All mice were held in individually ventilated cages (IVCs) in an SPF (Specific Pathogen Free) barrier unit. The animals were identified by numbered cages and by ear punches.

### Method Details

#### Dextran sodium sulfate challenge

A total of 10 mice were randomized into two treatment groups of five mice each. One group received no treatment and the other received 1.75% DSS (36–50 kDa MW, MP Biomedicals, lot #M9147) in their drinking water from study day 0 until mice were euthanized on the morning of study day 7. DSS was made fresh daily. Animal well-being was monitored once daily from day 0 until the end of the study. All mice were weighed and assessed for stool consistency and the presence of overt blood in the stool or around the anus. The scores were summed to calculate a disease activity index (DAI) for each mouse on the final study day (Figures S4A and S4B). Mice were euthanized by cervical dislocation on study day 7. The large intestine was removed and flushed with ice-cold Dulbecco’s (Ca^2+^& Mg^2+^ free) phosphate buffered saline (D-PBS, Sigma, UK) containing 100 U/mL penicillin and 100 μg/mL streptomycin (Sigma, UK). The length and wet weight of the large bowel were recorded prior to transferring the bowel to a 50 mL centrifuge tube, filled with RPMI 1640 medium with GlutamaxTM (Thermo Fisher, UK) and 100 U/mL penicillin and 100 μg/mL streptomycin. Large bowel samples were maintained at 2–6 ◦C and processed the same day.

For studying the efficacy of LOX family enzyme inhibition, three groups of male C57BL/6J mice were treated as follows: Two groups received 1.75% DSS in drinking water from day 0; the third group (control) remained untreated. β-aminopropionitrile (BAPN, 3-Aminopropionitrile fumarate salt, Sigma) was administered daily to one group of DSS recipients from study day 0 at 100mg/kg by intraperitoneal injection with the final administration on day 6. The other DSS-recipient group received test item vehicle (sterile saline) by the same regimen. Body weight and blood/stool observations were recorded daily. All procedures were certified according to the UK Animal (Scientific Procedures) Act 1986. Quantitative determination of thiobarbituric acid-reactive substances (TBARS) in plasma was performed at the conclusion of the experiment as a measurement of lipid peroxidation, which results in the formation of MDA. MDA reacts with thiobarbituric acid to form TBARS (TBARS Parameter Assay Kit, R&D Systems).

#### Human colonic stromal cell isolation

Up to eight endoscopic forceps biopsies were collected in DMEM supplemented with 100 U/ml penicillin, 100 μg/ml streptomycin and 10 mM HEPES on ice. Intact biopsies were incubated for 5 minutes at room temperature in ACK lysing buffer. After washing with PBS, the biopsies were dissociated using a human Umbilical Cord Dissociation Kit (Miltenyi Biotec) according to the manufacturer’s protocol for fresh tissue with some modifications. Incubation time was reduced to 2 hours and mechanical dissociation was achieved by passing the suspension through an 18 gauge needle 5–10 times every 60 minutes. The resultant cell suspension was passed through a 70 μm cell strainer and centrifuged at 500 G for 5 minutes. Depletion of non-stromal cell types was achieved by MACS separation following the manufacturer’s protocol with some modifications. The cell re-suspension buffer was substituted for HBSS with added penicillin 100 U/mL, streptomycin 100 μg/mL, HEPES 10 mM, EDTA 1 mM, and BSA 0.5% weight/volume. CD326 (EpCAM), CD45 and CD235a conjugated micro- beads were added to the cell suspension, and the mixture was incubated at 4°C for 15 minutes. Cells were then washed and loaded onto an LD column via a 35 μm pre-separation filter. The flow-through fraction was collected, and single cells pelleted by centrifugation at 500 G for 8 minutes.

#### Murine colonic stromal cell isolation

Colons were processed individually in parallel. Caecal pouches, mesenterium and fat were first detached and discarded. The remaining colon was opened longitudinally and cut into 1 cm fragments. These were incubated at 37 ◦C in RPMI with 0.1% BSA and 5 mM EDTA with horizontal shaking for 40 minutes to detach epithelial crypts. The crypt containing supernatant was discarded. Fresh RPMI with 0.1% BSA and 5 mM EDTA was added, and a further 15 minute incubation at 37 ◦C with horizontal shaking was performed to further deplete the epithelium. The tissue fragments were then washed and incubated in RPMI with added FCS (10%), HEPES (15 mM) and Collagenase VIII (100 U/mL, Sigma Aldrich) for 60 minutes at 37 ◦C with horizontal shaking. The resulting supernatant was passed through a 70 μm strainer and single cells were pelleted by centrifugation at 500 G for 8 minutes. Percoll gradient centrifugation was performed to remove non-cellular debris. Physiological 100% Percoll was made by combining 9 parts Percoll with 1 part 10X PBS. The cell pellets were resuspended in RPMI with 30% Percoll (GE Healthcare) and the resulting suspension layered over PBS with 70% Percoll in a 15 mL Falcon. Centrifugation at 900 G for 20 minutes (4 ◦C) was performed and the 30% / 70% interface layer was collected. MACS depletion of epithelial and hematopoietic cells was performed as for human stromal cells using in this case antibody-conjugated microbeads to murine EpCAM and CD45 (Miltenyi Biotec).

#### Single cell RNA sequencing, Fluidigm C1

Isolated single cells were re-suspended at a density of 700 live cells/μl in DMEM with 5% fetal calf serum (FCS). Cells were stained for DNA content and viability by supplementing the re-suspension buffer with NucBlue live cell stain (Life technologies, following the manufacturer’s protocol) and propidium iodide 10 μg/ml final. Cells were captured on the C1 system (Fluidigm) and processed using the SMARTer chemistry (Clontech) according to the Fluidigm protocol. External RNA Controls Consortium (ERCC) RNA spike-in mix was added to the lysis buffer 1:4000. C1 integrated fluidic circuits (IFCs) were imaged prior to cell lysis to identify sites occupied by single viable cells for downstream analyses. cDNA samples were selected after analyzing the cell images and prepared for sequencing using the Nextera XT library prep kit (Illumina). Libraries were sequenced using either Illumina HiSeq2500 100 bp paired-end sequencing or Illumina HiSeq4000 75 bp paired-end sequencing.

In experiments involving human donors with ulcerative colitis, biopsies from inflamed and non-inflamed bowel regions were collected separately, and single cell isolation was performed for both sets of biopsies in parallel. The resulting single cell suspensions were counter-stained with either CellTracker Green CMFDA or CellTracker Orange CMRA in addition to nuclear and viability staining with NucBlue live cell stain and Live/Dead fixable Far Red cell stain (all Life technologies). Counter-stained cell suspensions were mixed 1:1 immediately prior to loading onto the C1 IFC. Subsequent imaging and downstream processing was performed as above.

#### 10x library preparation and sequencing

Single cell suspensions were captured using the 10X Genomics® Chromium Single Cell 3′ Solution according to the manufacturers protocol. Cells from 3 DSS and 3 control mice were resuspendedseparately in PBS with 0.04% BSA at a density of 500 cells per μL. Murine RAW 264.7 macrophages stably transduced with a lentiviral expression construct containing an *Aspergillus fumigatus* blasticidin resistance gene and a monovalent citrine (mCitrine) yellow fluorescent protein (YFP) gene were spiked into the primary cell suspensions (5%). scRNA-seq libraries were generated using the Chromium Single Cell 3′ Reagent Kit v2 (10X Genomics). Briefly, a single-cell suspension in PBS with 0.04%BSA was mixed with RT-PCR master mix and loaded together with Single Cell 3′ Gel Beads and Partitioning Oil into a Single Cell 3′ Chip (10X Genomics) according to the manufacturer’s instructions. 3,500 cells were loaded into each reaction. RNA transcripts from single cells were uniquely barcoded and reverse-transcribed. cDNA molecules were pre- amplified fragmented, end repaired and ligated with Illumina adapters as per manufacturer’s protocol’ to generate a single multiplexed library that was sequenced over 3 Illumina HiSeq 4000 lanes. All libraries were quantified by Qubit and the size profiles of the pre-amplified cDNA and sequencing libraries were examined by the AATI Fragment Analyzer. For sequencing of human stromal cells, colonic biopsies were digested to obtain a single cell suspension (Umbilical Cord Dissociation Kit, human, Miltenyi). Undigested epithelial colonic crypts were removed by filtration, then the stromal fraction was enriched by MACS-depletion of CD45^+^, EpCAM^+^ and CD235a ^+^ cells. Simultaneous sample loading onto Chromium Single Cell A Chips was performed for each pair of healthy donor and patient samples. 7,000 cells were loaded for each set of reactions. Subsequent library generation was performed for all samples simultaneously as described above. Sample pooling was performed based on molarity and a single multiplexed library was sequenced over 4 Illumina HiSeq 4000 lanes.

#### Flow cytometry

Cells were extracted from biopsies obtained from healthy donors or ulcerative colitis patients with active inflammation. Sub-populations of cells were visualized using antibodies against cell surface markers (see Key Resources Table), then fixed and permeabilized using either the Foxp3 / Transcription Factor Staining Buffer Set (eBioscience) for detection of nuclear targets or Cytofix/CytopermTM (BD) for the detection of cytoplasmic targets. Intracellular targets were stained using either primary antibodies pre-conjugated using Lightning-LinkQR (Innova Bio- sciences) or a combination of primary antibody followed by secondary antibody staining (see Key Resources Table). For the detection of CCL19 and IL33, freshly-isolated cells were stimulated with PMA (0.2 μM) / ionomycin (1 μg/ml) for 4 hours in the presence of Brefeldin A (10 μg/ml) or in

Brefeldin A alone without stimulation, respectively. Where appropriate, an anti-fibroblast antibody (Miltenyi) was used for fibroblast visualization in combination with other cell surface markers, as expression of this antigen is not fibroblast-specific. Data were acquired on BD LSRII or BD Foretessa Instruments with FACS Diva. Data analyses were performed with FlowJo (Tree Star).

#### Mass cytometry time-of-flight

Lanthanide metal-labeled antibodies were obtained from Fluidigm or by conjugation of metal isotopes to purified antibodies using Maxpar Conjugation kits (Fluidigm). Biopsies from patients and healthy donors were digested using collagenase from Clostridium histolyticum (collagenase Type VIII, C2139, SIGMA) to obtain a single cell suspension after removal of undigested epithelial crypts by filtration. Cells were stained for surface markers followed by cisplatin staining was for dead cell exclusion, then cell fixation and permeabilization was performed using the Maxpar Nuclear Antigen Staining Buffer Set (Fluidigm). The Maxpar nuclear staining protocol was used for the simultaneous detection of cytoplasmic and nuclear targets. Iridium (191Ir) was used for cell visualization. Cells were acquired and analyzed on a CyTOF Helios mass cytometer and data were exported as FCS files. Gating was performed on single live CD45^-^EpCAM^-^CD31^-^ events for subsequent analyses of stromal events. Data analyses were performed using FlowJo (Tree Star).

#### Immunohistochemistry

Tissue slides were de-paraffinized, and antigen retrieval was performed by boiling slides for 20 minutes in citrate buffer, pH 6 in a vegetable steamer. Slides were allowed to cool, and endogenous peroxidase was blocked by incubating with 0.3% H_2_O_2_ in PBS for thirty minutes. Slides were washed in PBS and blocked with 2.5% Normal Goat Serum (Vector Laboratories, Peterborough, U.K) for thirty minutes. Slides were incubated with primary antibodies dissolved in 1% BSA in PBS and incubated for two hours at room temperature. Please refer to key resources table for antibodies used. Slides were washed in PBS containing 0.05% Tween 20 (PBS-T) and incubated with ImmPRESS HRP Anti-Rabbit or Anti-Mouse IgG (Peroxidase) Polymer Detection Kit (Vector Laboratories, Peterborough, U.K) for thirty minutes at room temperature. The slides were washed in PBS-T, and peroxidase activity was visualized using ImmPACT DAB Peroxidase (HRP) Substrate (Vector Laboratories). Finally, sections were counter-stained with Mayer’s hematoxylin, dehydrated and mounted. Primary antibody was excluded in the negative controls. For double staining, sections were de-paraffinized and antigen retrieval was performed by incubation for 20 minutes in citrate buffer, pH 6 in a vegetable steamer. Slides were allowed to cool and endogenous peroxidase was blocked by incubation with 0.3% H2O2 in distilled water for thirty minutes. Slides were washed with Tris-Hcl-buffered saline (TBS) and blocked with 2.5% Normal Goat Serum for thirty minutes. Sections were incubated with the first primary antibody for two hours. Thereafter, slides were washed with TBS and incubated for thirty minutes with ImmPRESSTM-AP Anti-Mouse or Anti-Rabbit IgG (alkaline phosphatase) Polymer Detection Kit (Vector Laboratories). Slides were washed in TBS containing 0.05% Tween20 for five minutes. AP activity was visualized with the AP substrate kit I Vector Red (SK-5100, Vector laboratories). Following this, the slides were washed in tap water and antigen retrieval was performed again for fifteen minutes in citrate buffer, pH6 in a vegetable steamer. The slides were subsequently blocked with Lab VisionTM Ultra V Block (Thermo Fisher Scientific, Paisley, UK) for fifteen minutes and incubated with the second primary antibody for two hours at room temperature. The slides were washed with TBS, incubated for thirty minutes with ImmPRESSTM-AP Anti-Mouse or Anti-Rabbit IgG (alkaline phosphatase) Polymer Detection Kit and AP activity visualized with AP substrate kit Vector Blue (SK-5300, Vector laboratories). The slides were washed in tap water and allowed to air dry before mounting in Vectamount (Vector laboratories). The quadruple staining was carried out as described by Van Der Loos (2010).

#### Single molecule RNA *in situ* hybridization

smISH was carried out on tissues that were fixed in 10% neutral buffered formalin for at least 24 hours on 5 μm sections. All probes and RNAscope 2.5 HD assay - brown (cat. 310035) were purchased from Advanced Cell Diagnostics (ACD, Milan, Italy) and used according to the manufacturer’s instructions. Probes used are listed in the key resources table, paraffin sections were pre-treated with Pretreat 1, 2, and 3 (all purchased from ACD). Pre-warmed (40 ^◦^C) probes were added to the slides and incubated in the HybEZ oven (catalog 321461; ACD) for 2 hours at 40°C. After a 6-step signal amplification, tissues were detected by DAB (all part of the RNAscope 2.5 HD assay - brown kit) and counter-stained with Mayer’s hematoxylin. Slides were mounted with PERTEX mounting medium (Gothenburg, Sweden) and photographed.

#### Organoid cultures from human colonic crypts

Organoid cultures were established as originally described by Sato et al. (Sato et al., 2011). Briefly, cultures were established from eight pairs of colonic biopsies. Single cell suspensions were obtained by incubation with 0.4mg/mL Dispase (GIBCO). Crypts were mixed with 50uL Matrigel (Corning) and plated on pre-warmed 24-well culture dishes. Embedded cells were overlaid with WREN medium (Wnt3a conditioned medium (L Wnt¬3A (ATCC® CRL¬2647TM)) and ADF (Advanced DMEM-F12 medium - GIBCO) 50:50, Glutamax (Life Technologies), 10mM HEPES, N-2 [1x] (Life Technologies), B-27 [1x] (Life Technologies), 10mM Nicotinamide (Sigma Aldrich), 1mM N-acetyl-L-cysteine (Sigma Aldrich), 1ug/ml R-spondin 1 (RSPO1) (Peprotech), 50ng/mL human epidermal growth factor [EGF] (Peprotech), 100ng/mL human Noggin (Peprotech), 1ug/mL Gastrin (Sigma Aldrich), and 0.05uM PGE2 (Sigma Aldrich), 0.1uM A83-01, 10uM p38 inhibitor SB202190, 10uM Y27632 (all from R&D Systems). Medium was replaced with fresh WREN medium every other day.

For co-culture experiments, intestinal stromal cells were isolated from fresh biopsies as previously described. Following appropriate antibody staining (CD45 AF700, CD142(F3) APC, Cd326(EpCam) PE, CD31 FITC, DAPI) and compensation control (CompBeads/CompBeads Plus, BD) samples were sorted in sterile conditions using a FACS Aria III (BD) with populations defined as F3^+^ (live, CD45^-^EpCam^-^CD31^-^F3^high^) or F3^-^ (live CD45^-^EpCam^-^CD31^-^F3^low^). We mixed a total of 200 crypts with 2 × 10^4^ F3^+^ or F3^-^ cells and embedded them in Matrigel. After polymerization, we added modified crypt medium (without Wnt) composed of Advanced DMEM/F12 supplemented with 10% (vol/vol) bovine serum, 100U/mL penicillin/streptomycin, 10mM HEPES, 1x N2, 1x B27, 50ng/mL EGF, 100ng/mL Noggin and 500ng./mL RSPO1. We followed the culture over a course of 11 days with images acquired every day. ‘Remarkable’ debris were used as tracking landmarks to identify each organoid imaged.

For quantitative RT-PCR experiments, we isolated RNA from organoids grown in WREN medium and stimulated for four days with either 100ng/mL of Interleukin-6 (R&D Systems) or 100ng/mL of TNFSF14/LIGHT (R&D Systems). In indicated experiments, we cultured them for four days as above and then withdrew WNT by changing the medium to one without any WNT, Nicotinamide and SB202190 for four more days with either IL-6 or LIGHT.

#### Assessing Organoid proliferation

EdU staining to analyze cell proliferation was performed using the Click-iT EdU Alexa Fluor 647 imaging kit (Invitrogen). Organoids were incubated with 5 μM EdU for 6 hr followed by fixation for 20 min with 4% para-formaldehyde. The Click-iT reaction cocktail was added according to manufacturer’s protocol and incubated for 30 min. Nuclear stain (DAPI 1ug/ml - Sigma Aldrich) was added for 5 min. Whole mount images were obtained via z stack reconstruction using the Leica SP-8. Quantification presented as the percentage of EdU positive cells relative to the total number of nuclei counted. A minimum of 500 cells from 15 images in 3 independent experiments are presented.

#### Organoid RNA isolation and quantitative RT-PCR

For RNA isolation, organoids were harvested by dissolving Matrigel including organoids with ice-cold PBS. Following centrifugation at 300*g* for five minutes at 4°C, the supernatant was discarded and the pelleted organoids were resuspended in 350uL of RLT buffer (QIAGEN). Total RNA was isolated using the RNeasy Mini Kit (QIAGEN) according to the manufacturer’s instructions. cDNA was synthesized using iScript cDNA Synthesis Kit (Bio-Rad) following the manufacturer’s instructions. Quantitative RT-PCR was performed using TaqMan® gene expression assays. Taqman Gene Expression assays used are listed in the key resources table.

### Quantification and Statistical Analysis

#### Bioinformatic approach, Fluidigm C1

##### Alignment and transcriptome assembly

Reads were demultiplexed using the index barcodes to generate single cell libraries. Adaptor sequences (IlluminaP7, Nextera, SMARTer) were trimmed using the Skewer package (Jiang et al., 2014), then aligned to an Ensembl GRCh38 human genome index with HISAT (Kim et al., 2015). Sorting and duplicate read detection was performed using Picard Tools (http://broadinstitute.github.io/picard/) prior to summarization with featureCounts (Liao et al., 2014) using Ensembl Release 84 (March 2016) transcriptome annotation. Reads flagged as optical duplicates, multi-mapping and ambiguously mapping reads were discarded.

##### Cell and gene QC

Raw sequence QC reports were generated with FastQC using default settings (http://www.bioinformatics.babraham.ac.uk/projects/fastqc/). These and other QC outputs generated by the pipeline tools above were collated using multiQC, imported together with gene count tables into R, and assembled into an SCESet object using the package scater (McCarthy et al., 2017). Single cell libraries were assessed against a panel of QC metrics including total reads aligning to genomic features, number of unique genes detected, sequencing saturation, the proportion of reads mapping to ERCC controls, and the proportion of reads mapping to mitochondrial features. Libraries performing poorly across these metrics, with reference to bulk and empty capture site controls where appropriate, were removed from the dataset.

##### Library size normalization

Library size normalization was performed first by using the R package ‘scran’ to generate cell-based scaling factors (Lun et al., 2016). Normalized read counts were converted to variance stabilized expression values by log2 transformation with an offset of 1. A mean-variance trend was then fitted to the expression values. Control genes were identified *in silico* as genes with total variance below the fitted value. These genes were considered to show only technichal variability and were used to perform a further normalization step using the ‘RUVg’ method from the R package ‘RUVseq’ (Risso et al., 2014).

##### Weighted gene co-expression network analysis

Gene co-expression network construction was performed in R using the package ‘WGCNA’ (Langfelder and Horvath, 2008). An expression matrix of biologically variable genes and cells passing QC was converted to a pairwise gene adjacency matrix by Pearson correlation. Negative correlations were set to zero (a ‘signed-hybrid’ network). This was, in turn, converted to a dissimilarity matrix by subtraction from 1 and a soft- thresholding power of 4 was applied. As a further noise-filtering step, an expression level threshold was set that represented ‘high confidence’ detection of a gene, and pairwise connectivities based on few cells with ‘high confidence’ detection of both genes were removed from the network prior to clustering and module detection. The ‘high confidence’ thresholds used were between 5 and 8 normalized counts while the minimum cell number was 3 or 4 depending on the dataset. Genes remaining in the filtered dissimilarity matrix were hierarchically clustered by average linkage, and the resulting clustering tree was cut to find co-expression modules using the ‘cutreedynamic’ function with a deep split setting of 3 and a minimum module size of between 30 and 50 genes.

##### Cell clustering

Following module detection, a matrix comprising expression values for all genes assigned to modules was used as a basis for cell clustering by non-negative matrix factorisation using the R package ‘NMF’ (Gaujoux and Seoighe, 2010). 50 randomly initiated runs were performed to generate a consensus clustering matrix with values between 1 (cells always in the same cluster) and 0 (cells never in the same cluster). We surveyed a range of possible rank values, calculating cophenetic correlation, average silhouette width, factor dispersion, and RSS, seeking the first local maximum in the first three measures and the inflection point in the last. The same metrics were calculated for randomized data by independent permutation of the gene expression values as a control. The optimal rank values of 4 and 5 were used for the healthy and UC datasets respectively.

##### Marker genes and ontology enrichment

For each gene, mean expression levels were calculated in each cell cluster, and a binary classifier was constructed to test the ability of that gene to distinguish cells in the cluster with the highest mean expression from the remaining cells. This was quantified in terms of the area under the ROC curve. The top 200 genes for each cluster, ranked in this way, were selected as candidate marker genes. Biological process GO enrichment was performed both on detected gene modules and cluster marker genes using a hyper-geometric test with correction for multiple testing using the Benjamini & Hochberg (BH) method with both p value threshold and false discovery rate set to 0.05. Dot and network plots were produced with the package ‘clusterProfiler’ in R (Yu et al., 2012).

##### Reduced dimensionality representations

Principle component analysis (PCA) was performed using the function ‘prcomp’ in R. T-distributed stochastic neighbor embedding (t-SNE) was performed using the package ‘Rtsne’ with an initial PCA step. Ten randomly initiated t-SNE runs were performed and the solution with the lowest Kullback-Leibler divergence was selected for visualization.

#### 10x genomics computational analysis

##### Cellranger Pipeline

The Cell Ranger version 2.1.1 software suite was obtained from 10x Genomics (https://support.10xgenomics.com/single-cell-gene-expression/software/downloads/latest). Raw sequencing data was first de-multiplexed using the Illumina bcl2fastq software to generate separate paired-end read files for each sample, which were quality-checked using FastQC software. For murine sample libraries, alignment and transcript quantification was performed with the standard Cell Ranger ‘count’ script against a custom genome reference incorporating the University of California Santa Cruz (UCSC) mm10 murine genome assembly and the lentiviral construct present in the control cells. Replicate control and DSS samples were then aggregated using the Cell Ranger ‘aggr’ script with the default normalization step (by down sampling) disabled. Similarly, Cell Ranger ‘count’ script was also used to align human fastq files to the human hg38 assembly analysis set reference genome (UCSC). UMI counts were summarized using Ensembl gene annotation GTF file obtained using the UCSC Table Browser tool. Replicate HC and UC samples were aggregated using the Cell Ranger ‘aggr’ script as described before. The raw (unfiltered) count matrices of both human and mouse data were imported into R for further processing.

##### Identification of cellular barcodes and QC

Raw expression matrices output from the Cell Ranger pipeline were first filtered to remove barcodes with fewer than 250 unique molecules detected. A density plot of total unique molecular identifiers (UMIs) was then constructed for the remaining barcodes (Figure S6A). This revealed a bimodal distribution with the higher peak representing Gel Beads in emulsion (GEMs) that successfully captured cells. The UMI threshold was set at the first local minimum on the density plot for each sample and barcodes with fewer UMIs were removed. In addition to cells with low total UMI counts, cells with high percentage of UMIs (> 5%) originating from mitochrondrial features were also filtered out at this stage.

Initial clustering was performed using the R package ‘Seurat’ (Version 2.3.0) (Satija et al., 2015). Low-expressed genes (detected in fewer than 3 cells) were first removed, then variable genes were annotated by examination of the mean-variance relationship. Variable genes met the following criteria: 0.0125 < mean of non-zero values < 4 AND standard deviation > 0.5. Dimensionality reduction was then performed using PCA. In mouse data, the first 10 principle components were used to generate an initial clustering using the Seurat community detection algorithm to identify the control cell cluster. Control cell cluster was detected based on expression of the lentiviral blasticidin resistance gene and known macrophage markers and was excluded from subsequent analyses.

##### Cell cycle annotation and clustering

Cell cycle stage annotation was performed using the ‘cyclone’ function from the R package ‘scran’, (Version 1.6.9), which implements a previously published robust gene-pair based prediction method (Scialdone et al., 2015). Unwanted variation due to library size (total UMIs), experimental batch and the G1 and G2M cell cycle scores was then regressed out. PCA was repeated using the adjusted expression values. The number of principle components used as a basis for clustering was determined by examining the scree plot and the statistical jackstraws test, which determined the robustness of a principle component by repeated permutation of small fractions of the dataset. All significant principal components detected by the jackstraw test were used to generate final Seurat clusters and accompanying t-SNE plots for visualizations.

##### Random Forest Models

Random Forest models were trained in R using ‘randomForest’ R package (Liaw, 2002) with scaled and normalized gene expression values (see above, Seurat methods) for each gene as training features and the following parameters: ntree = 1000; mtry = square root of total genes in each model. Feature selection was performed by recursive feature elimination using ‘caret’ R package (Kuhn, 2008) with 10-fold cross-validation using mouse or human gene expression as training data for each respective model. Due to unbalanced class distribution in human scRNA-seq data, prior to feature selection and model training, cells from human S1 and S2 clusters were down-sampled and cells from human S4 cluster were up-sampled using SMOTE algorithm (Torgo, 2010) to obtain a balanced training dataset. SMOTE implementation in ‘DMwR’ r package was used with the nearest neighbor parameter k = 5 for generating new minority class observation examples.

For cross-species Random Forest models, the data were pre-processed as follows. Human-mouse gene orthologs were obtained from Ensembl using the Biomart tool, and genes were filtered to remove genes with ‘one-to-many’,’ many-to-many’ and ‘many-to-one’ relationships. The remaining gene orthologs were further filtered to keep only mappings with minimum 75% sequence identity between pairs. All high confidence orthologs detected with at least minimal expression across both human and mouse scRNA-seq data were then used to model feature selection. Model classification performance was assessed using ‘pROC’ R package (Robin et al., 2011) by computing Area Under Receiver Operating Curve (AUC) and examination of confusion matrices. Individual model feature cluster specificities were computed as AUC scores using ‘Seurat’ R package (Satija et al., 2015).

##### Further Seurat analysis functions

Hierarchical phylogenetic trees were constructed using the ‘BuildClusterTree’ function. Out of bag error rates (OOBEs) for internal nodes in the tree, representing the accuracy with which cells could be allocated to branches by a random forest classifier, so cluster confidence was generated using the ‘AssessNodes’ function. Cluster marker genes were identified using ‘FindMarkers’ and ‘FindAllMarkers’ functions with the following additional settings: min.pct = 0.25, thresh.use = 0.25, return.thresh = 0.3, test.use = ‘roc’. Cluster-specific differentially expressed genes comparing the mouse DSS and control datasets and human datasets were generated with the ‘FindMarkers’ function using a negative binomial differential expression test (test.use = ‘negbinom’) and the total UMI counts, G1 and G2M cell cycle scores and batch annotation as latent variables.

##### Batch Effect Assessment

To ensure that clustering was not driven by batch effects, batch distributions for each dataset were visualized using tSNE plots (Figure S6C). For each dataset, we also computed entropy of batch mixing, as described by (Haghverdi et al., 2018), for tSNE cell embeddings of sample batches. As a negative control (no batch effect), we assigned each cell a random batch label and computed the expected entropy. Similarly, as a positive control (clustering is driven entirely by batch effects), we used cluster identities as batch labels for entropy calculations. Each set of entropies was computed from the neighborhoods of 100 randomly picked cell locations, bootstrapped 100 times and the distributions visualized as boxplots (Figure S6B).

#### Ontology Enrichment Analysis

GO enrichment of cluster markers and differentially expressed genes was performed using the R package ‘clusterProfiler’ (Yu et al., 2012) with a Benjamini-Hochberg multiple testing adjustment and a false-discovery rate cut-off of 0.05, using all expressed genes within human or mouse data respectively as background control. The results were visualized using ‘clusterProfiler’ ‘dotplot’ function, and ‘ggplot2′ and ‘igraph’ packages.

#### Comparison with FRCs

Microarray expression data from blood endothelial, lymphatic endothelial, skin fibroblasts, thymus fibroblasts, fibroblastic reticular cells and contractile double-negative cells were downloaded from GEO (GSE15907) as RMA normalized signal intensity matrices. Microarray probes were mapped to mouse gene symbols and probes with many-to-one and many-to-many relationships were filtered out. Cell clusters identified from DSS mouse scRNA-seq data were combined into ‘pseudo-bulk’ sets for Endothelial, Lymphatic, Pericyte, Myofibroblast and Stromal sub-type cells. To facilitate a degree of comparability between microarray expression intensities and single cell clusters, quantile normalization, as implemented in R package ‘preprocessCore’ was performed. The pericyte “pseudo-bulk” cluster was used as the reference sample for normalization, as these cells represented the smallest cell cluster. Following quantile normalization, batch correction was performed using ‘ComBat’ algorithm implemented in R package ‘sva’ (Johnson et al., 2007). Hierarchical clustering (complete linkage) was performed in R using ‘hclust’ function, using all cluster marker genes detected as described previously.

#### Diffusion maps and diffusion pseudotime

Diffusion maps and diffusion pseudotime analysis was performed with the R package ‘destiny’ (Angerer et al., 2016). An expression matrix consisting of normalized variable gene UMI counts for cells annotated to fibroblast clusters in the control dataset (Str 1.1, 1.2, 1.3, 2, and 3) was input to the function ‘DiffusionMap’ and a diffusion map generated with a local scale parameter (sigma = ‘local’), rotated eigenvalues (rotate = TRUE) and considering each cell’s 500 nearest neighbors (k = 500). Diffusion pseudotime was calculated using the function ‘DPT’ with default settings.

### Data and Software Availability

The accession numbers for the raw and processed data files reported in this paper are GEO: GSE95459 and GSE114374. Analysis scripts for scRNA-seq data from 10x genomics libraries are available at: https://github.com/agneantanaviciute/colonmesenchymescrnaseq

## Supplemental Information

Table S1. Mesenchymal Cells Used in scRNA-Seq Analyses, Related to Figures 1–4

Table S2. Cluster Marker Genes in Health, C1 Data, Related to Figure 1

Table S3. Cluster Marker Genes in Health, 10x Data, Related to Figure 1

Table S4. Differentially Expressed Genes between S2a and S2b Sub-clusters, Related to Figure 1

Table S5. Cluster Marker Genes in UC, C1 Data, Related to Figure 2

Table S6. Cluster Marker Genes in Mouse in Health, Related to Figure 3

Table S7. Cluster Marker Genes in DSS-Treated Mouse, Related to Figure 4

Table S8. Differentially Expressed Genes between Clusters in Healthy and DSS-Treated Mice, Related to Figure 4
