# Supplementary material for: Structural Remodeling of the Human Colonic Mesenchyme in Inflammatory Bowel Disease
Source: Cell. 2018 Oct 4;175(2):372–386.e17. doi: 10.1016/j.cell.2018.08.067 (PMC6176871; doi:10.1016/j.cell.2018.08.067)
Supplement: Table S1. Mesenchymal Cells Used in scRNA-Seq Analyses, Related to Figures 1–4 [file mmc1.pdf]

Cell, Volume *175*

Supplemental Information

Structural Remodeling of the Human Colonic Mesenchyme in Inflammatory Bowel Disease

James Kinchen, Hannah H. Chen, Kaushal Parikh, Agne Antanaviciute, Marta Jagielowicz, David Fawkner-Corbett, Neil Ashley, Laura Cubitt, Esther Mellado-Gomez, Moustafa Attar, Eshita Sharma, Quin Wills, Rory Bowden, Felix C. Richter, David Ahern, Kamal D. Puri, Jill Henault, Francois Gervais, Hashem Koohy, and Alison Simmons

Table S1: Mesenchymal cells used in scRNA-Seq analyses. Related to Figures 1-4.

Sample	Species	Type	Number of Cells	Technology	Gender	Age
S1	Human	Healthy	57	C1 Fluidigm/Smart-Seq2	F	69
S2	Human	Healthy	58	C1 Fluidigm/Smart-Seq2	F	57
S3	Human	Healthy	68	C1 Fluidigm/Smart-Seq2	F	50
S4.1	Human	UC non-inflamed	13	C1 Fluidigm/Smart-Seq2	M	29
S5.1	Human	UC non-inflamed	31	C1 Fluidigm/Smart-Seq2	M	45
S6.1	Human	UC non-inflamed	9	C1 Fluidigm/Smart-Seq2	M	30
S4.2	Human	UC inflamed	24	C1 Fluidigm/Smart-Seq2	M	29
S5.2	Human	UC inflamed	19	C1 Fluidigm/Smart-Seq2	M	45
S6.2	Human	UC inflamed	31	C1 Fluidigm/Smart-Seq2	M	30
S7	Human	Healthy	1919	10x Genomics	F	65
S8	Human	Healthy	2459	10x Genomics	M	55
S9	Human	UC inflamed	2097	10x Genomics	F	35
S10	Human	UC inflamed	2806	10x Genomics	F	44
S11	Mouse	Healthy	1258	10x Genomics	M	10-12 weeks
S12	Mouse	Healthy	1244	10x Genomics	M	10-12 weeks
S13	Mouse	Healthy	1258	10x Genomics	M	10-12 weeks
S14	Mouse	DSS	1394	10x Genomics	M	10-12 weeks
S15	Mouse	DSS	909	10x Genomics	M	10-12 weeks
S16	Mouse	DSS	1188	10x Genomics	M	10-12 weeks
